# Synthesis of novel coumarin–hydrazone hybrids as α-glucosidase inhibitors and their molecular docking studies†

**DOI:** 10.1039/d3ra03953f

**Published:** 2023-09-04

**Authors:** Hafiza Zara Tariq, Aamer Saeed, Saeed Ullah, Noor Fatima, Sobia Ahsan Halim, Ajmal Khan, Hesham R. El-Seedi, Muhammad Zaman Ashraf, Muhammad Latif, Ahmed Al-Harrasi

**Affiliations:** a Department of Chemistry, Quaid-i-Azam University Islamabad 45320 Pakistan asaeed@qau.edu.pk +92-51-9064-2128; b Natural and Medical Sciences Research Center, University of Nizwa P.O. Box 33, PC 616, Birkat Al Mauz Nizwa Sultanate of Oman aharrasi@unizwa.edu.om; c School of Food and Biological Engineering, Jiangsu University Zhenjiang 212013 China; d Department of Chemistry, Faculty of Science, Menoufia University Shebin El-Kom 32512 Egypt; e Department of Chemistry, Allama Iqbal Open University Islamabad Pakistan; f Centre for Genetics and Inherited Diseases (CGID), Taibah University Al-Madinah Al-Munawwarah Kingdom of Saudi Arabia

## Abstract

Diabetes mellitus is a metabolic disorder and more than 90% of diabetic patients suffer from type-2 diabetes, which is characterized by hyperglycemia. α-Glucosidase inhibition has become an appropriate approach to tackle high blood glucose levels. The current study was focused on synthesizing coumarin–hydrazone hybrids (7a–i) by using facile chemical reactions. The synthesized compounds were characterized by using ^1^H-NMR, ^13^C-NMR, and IR. To evaluate their anti-diabetic capability, all of the conjugates were screened for *in vitro* α-glucosidase inhibitory activity to reveal their therapeutic importance. All of the compounds (except 7b) demonstrated significant enzyme inhibitory potential with IC_50_ values ranging between 2.39–57.52 μM, as compared to the standard inhibitor, acarbose (IC_50_ = 873.34 ± 1.67 μM). Among them, compound 7c is the most potent α-glucosidase inhibitor (IC_50_ = 2.39 ± 0.05 μM). Additionally, molecular docking was employed to scrutinize the binding pattern of active compounds within the α-glucosidase binding site. The *in silico* analysis reflects that hydrazone moiety is an essential pharmacophore for the binding of compounds with the active site residues of the enzyme. This study demonstrates that compounds 7c and 7f deserve further molecular optimization for potential application in diabetic management.

## Abstract 2

This study introduces novel coumarin–hydrazone hybrids, assessed for their potential as α-glucosidase inhibitors to treat diabetes. Compounds checked for α-glucosidase inhibition and *in silico* docking was also carried out. Studies suggest promising therapeutic significance and pharmaceutical use.

## Graphical abstract

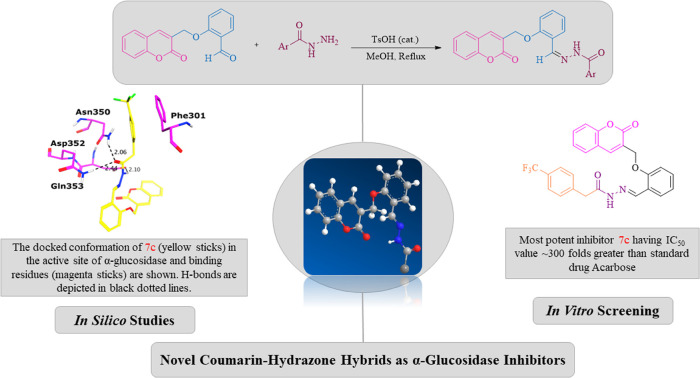

## 1. Introduction

Coumarins or 2*H*-chromen-2-ones are heterocyclic compounds with extensive biological profiles.^1^ These structurally bicyclic motifs comprise a benzene ring fused with a 2-pyrone ring at its position 5 and 6.^2^ Coumarins constitute a large class of secondary metabolites and their natural occurrence is spanned over several decades.^3,4^ The therapeutic properties of coumarins are noteworthy and have led to the development of versatile methods to synthesize these compounds in the laboratory. Consequently, numerous structurally variant coumarins have been formulated to take utmost advantage of their biological importance.^5^

Molecules incorporating coumarins have been shown to possess profound biological activities including anti-convulsant,^6,7^ anti-diabetic,^8^ anti-coagulant,^9^ antioxidant,^10^ anti-Alzheimer's,^11,12^ anti-cancer,^13^ anti-proliferative,^14^ anti-bacterial,^15–17^ anti-fungal^18^ and anti-viral^19^ properties. Considering the numerous pharmacological activities, low molecular weight, high bioavailability and low toxicity of coumarin analogues, these scaffolds have sheer importance in drug design and medicinal chemistry as unique pharmacophores.^2,4^

Hydrazones belong to the class of azomethine compounds with C=N linkages and are related to the category of Schiff bases.^20^ Hydrazones possess two interlinked nitrogen atoms, while Schiff bases have an alkyl or aryl group directly attached to the nitrogen of the azomethine group.^21^ In other words, hydrazones are Schiff bases of hydrazine or hydrazides. Hydrazones find immense pharmaceutical usage because of their biological activities like anti-inflammatory,^22^ anti-cancer,^23^ anti-tyrosinase,^24^ anti-convulsant,^25^ antioxidant,^26^ anti-fungal^27^ and anti-bacterial propensity.^28^

Diabetes mellitus is a metabolic disorder and is associated with a decrease in insulin action or its production. Currently, more than 90% of diabetic patients suffer from type-2 diabetes globally. This form of diabetes is characterized by insulin resistance and hyperglycemia.^29,30^ α-Glucosidase inhibitors are clinically approved class of drugs that improve glycemic index in diabetic patients.^31,32^ α-Glucosidase is a digestive enzyme in the small intestine that breaks down disaccharides and oligosaccharides into monosaccharides. As a result, it increases the blood glucose levels.^33^ α-Glucosidase inhibitors help to alleviate the blood glucose levels in diabetes type-2 patients, mitigating postprandial hyperglycemia. Several molecules like acarbose, miglitol, and voglibose are already available drugs in market for diabetes that target α-glucosidase enzyme, however these molecules have side effects including diarrhea, cramping, and colonic gas production and they are also expensive.^34,35^ Therefore, the α-glucosidase inhibitors present a fascinating pathway to upgrade treatment approaches and allow individuals with type 2 diabetes to achieve precise control over their blood sugar levels. This has urged scientists to make diverse sets of α-glucosidase inhibitors.^36,37^

Coumarins have been hybridized with isatin I^36,38^ and dithiocarbamate II^39^ in 2017 and 2021, respectively, and those molecules showed excellent inhibition of α-glucosidase enzyme. Similarly, hydrazone derivatives III^40^ and IV^41^ are two of the prominent inhibitors of α-glucosidase as demonstrated by studies. Recent studies have confirmed coumarin–hydrazone conjugates as α-glucosidase inhibitors. The coumarin–iminothiazolidinone hybrid V exhibited remarkable enzyme inhibition as compared to acarbose which is usually used as a reference drug in the *in vitro* tests.^42^ Likewise, Taha *et al.* in 2018 synthesized coumarin-based hydrazone which showed inhibitory potential for α-glucosidase enzyme with IC_50_ value of 1.10 ± 0.01 μM as compared to standard acarbose.^29^ In 2020, a group of scientists formulated a novel series of phthalimide-Schiff base-coumarin hybrids with α-glucosidase inhibitory activity which shows compound VI as one of the exceptionally active compounds as compared to acarbose.^43^

**Fig. 1:**
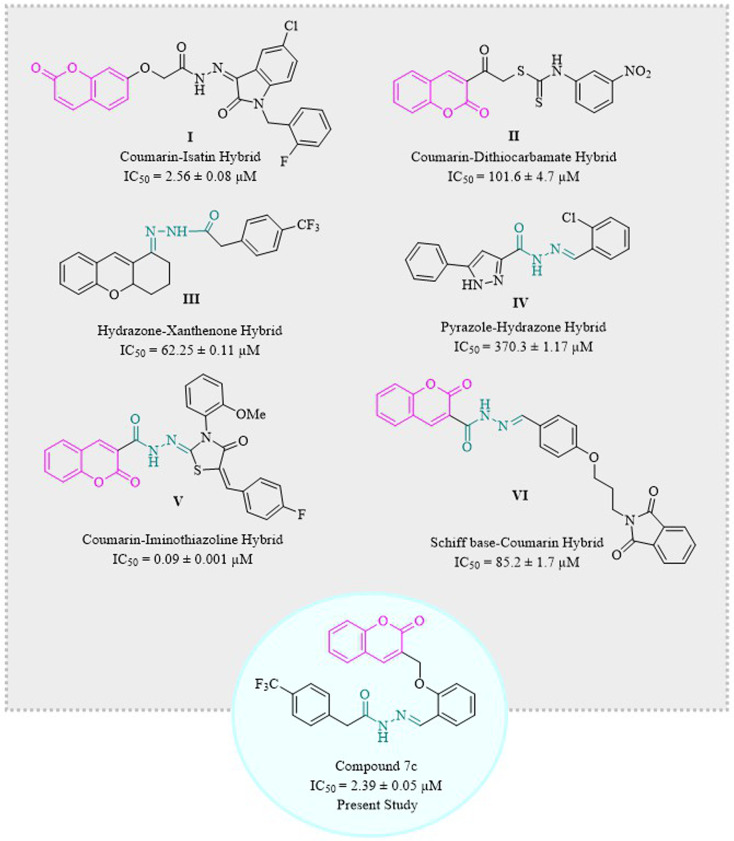
Some recent examples of coumarin and hydrazones as α-glucosidase inhibitors.

Designing and synthesizing conjugates of already available medicinally important compounds helps in finding new lead compounds. The biological and pharmacological importance of coumarins and hydrazones encouraged us to synthesize a series of new and novel derivatives of 2*H*-chromen-2-one tethered hydrazones. Herein, we are reporting the synthesis, characterization, and α-glucosidase inhibition activity of structurally and chemically variable coumarin–hydrazone hybrids (Fig. 1).^39–44^ Some other compounds are also reported for their *in vitro* α-glucosidase activity like β-carboline derivatives, and betulinic acid derivatives.^45,46^ Hence, the recent study was designed to evaluate the anti-diabetic potential of synthetic coumarin–hydrazone hybrids.

## 2. Results and discussion

### 2.1. Chemistry

**Scheme 1:**
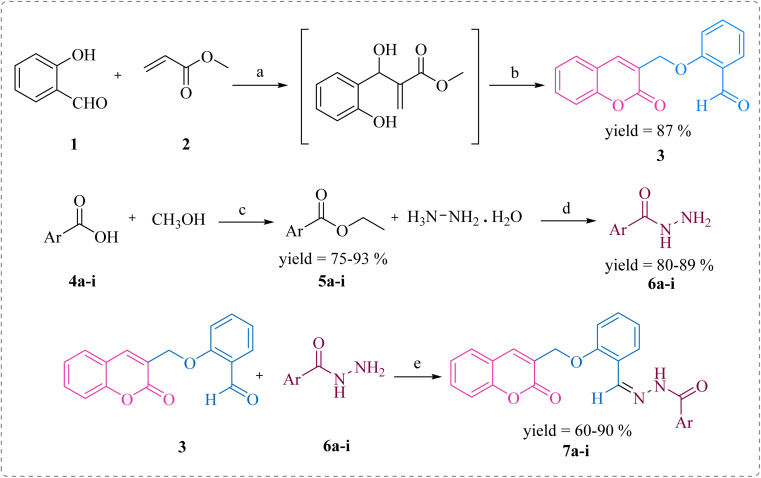
Synthesis of compounds 7a–i. Reagents and conditions: (a) (C_2_H_5_)_3_N, dry MeOH, rt; (b) rt, 15–18 h; (c) H_2_SO_4_, MeOH, reflux, 15 h; (d) MeOH, reflux, 3–4 h; (e) TsOH, dry MeOH, reflux, 5–6 h.

In this study, the coumarin–hydrazone hybrids (7a–7i) were synthesized as per the route illustrated in Scheme 1. The generation of unique conjugates consists of a single step reaction involving Schiff base condensation between coumarin-based aldehyde and a series of acid hydrazides. However, both the reagents for this single step reaction were synthesized separately. By stirring readily available salicylaldehyde (1) with methyl acrylate (2) and triethylamine in methanol as a solvent for 18 hours at room temperature, 2-((2-oxo-2*H*-chromen-3-yl)methoxy)benzaldehyde (3) was obtained in 87% yield. This product was formed *via* Baylis Hillman strategy followed by cyclization reaction. The substituted esters (5a–i) were made by reacting acids (4a–i) with methanol in presence of sulfuric acid catalyst under reflux. Consequently, the substituted acid hydrazides (6a–i) were generated by reaction of respective ester (5a–i) and hydrazine, in dry methanol under reflux in presence of catalytic amount of *p*-toluene sulphonic acid. This reaction took place following the nucleophilic acyl substitution. The acid hydrazides obtained were purified by recrystallization in ethanol. Followed by the formation of substituted acid hydrazides, condensation reactions between coumarin-based aldehyde and hydrazides were conducted to yield the coumarin–hydrazide conjugates *i.e.*, hydrazones 7a–i.

The ^1^H-NMR and ^13^C-NMR spectra of compounds confirmed the formation of all conjugates. In case of 7c, ^1^H-NMR shows singlet at *δ* 12.02 for N–H and at *δ* 8.84 for azomethine proton, besides the methylene group at *δ* 3.32. ^13^C signals at *δ* 144.3 corresponded to C=N of azomethine, 159.6 for C=O of coumarin. Similarly, the methylene carbon resonates at 65.5 ppm. However, in the case of 7a and 7i, the signals for N–H appear as doublet. It might be due to ^4^*J* coupling of the respective protons.

### 2.2. α-Glucosidase inhibitory activity and structure–activity relationship (SAR) analysis

In order to understand the intricate details of the structure–activity relationship, a series of coumarin-based hydrazones was made *via* molecular hybridization strategy. The synthesized derivatives were then screened for *in vitro* α-glucosidase inhibitory activity and their activity was compared with the commercially marketed drug, acarbose (Table 1).

**Table 1:** α-Glucosidase inhibitory activity of coumarin–hydrazone conjugates^a^

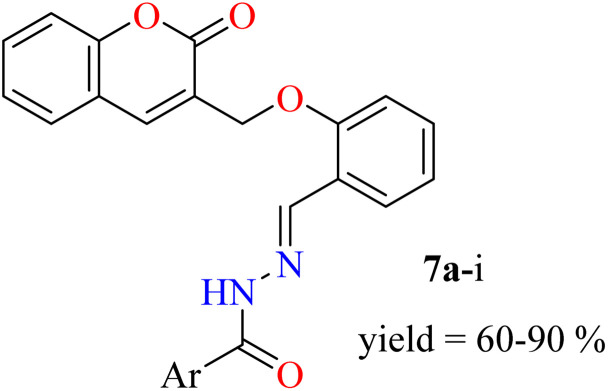			
Compounds	Ar-group	Percent inhibition (0.5 mM) ± SEM	IC_50_ ± SEM (μM)
7a	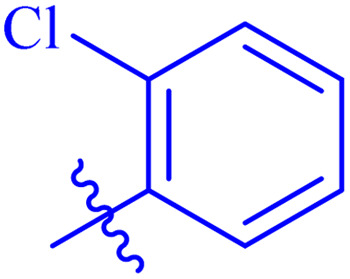	93.64 ± 0.58	4.95 ± 0.17
7b	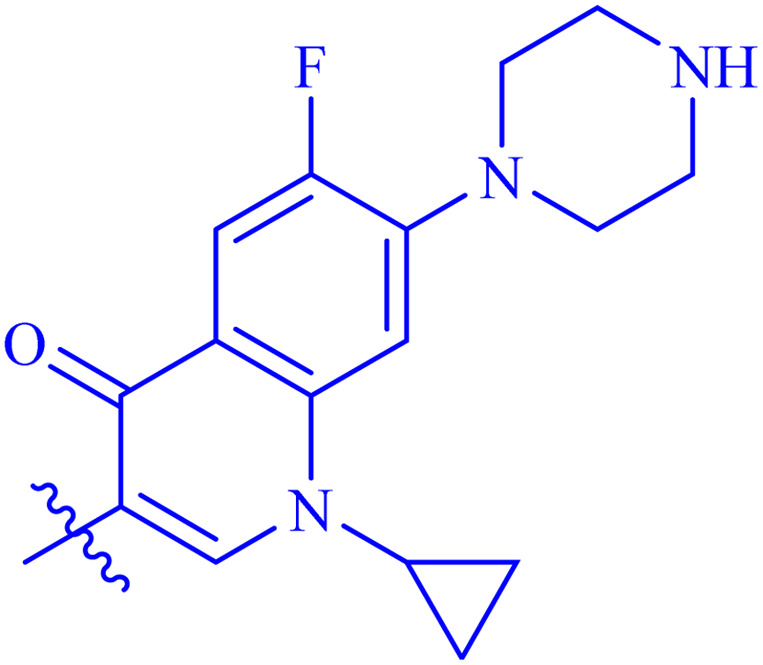	39.86 ± 0.62	N/A
7c	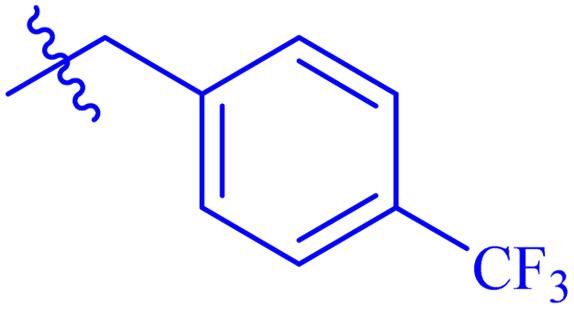	94.27 ± 0.41	2.39 ± 0.05
7d	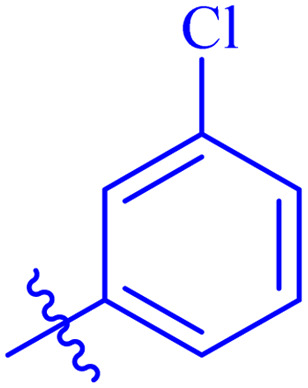	94.18 ± 0.36	3.76 ± 0.14
7e	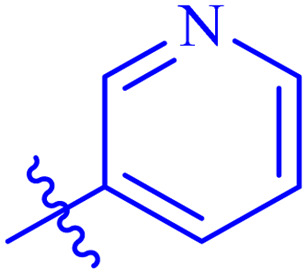	78.90 ± 0.63	57.52 ± 0.43
7f	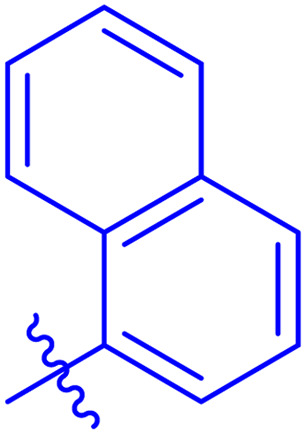	94.71 ± 0.37	2.90 ± 0.06
7g	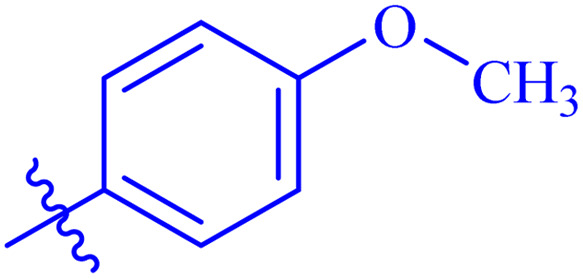	93.59 ± 0.40	5.81 ± 0.12
7h	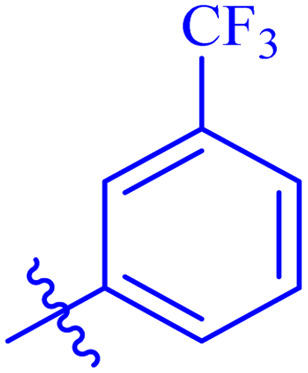	92.63 ± 0.35	6.14 ± 0.13
7i	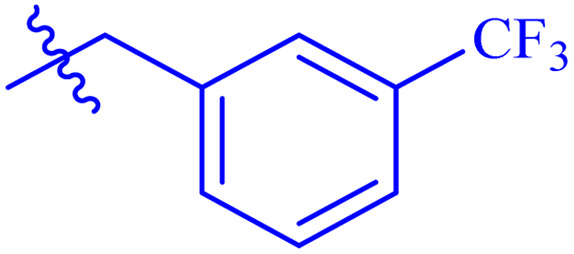	92.47 ± 0.61	17.90 ± 0.24
Standard (acarbose)	—	59.37 ± 0.83 (1 mM)	873.34 ± 1.67

a SEM (Standard Error Mean), N/A (Not Active).

All the derivatives (except 7b) exhibited remarkable α-glucosidase inhibitory activity with IC_50_ values in the range of 2.39 ± 0.05–57.52 ± 0.43 μM, as compared to the standard inhibitor, acarbose (IC_50_ = 873.34 ± 1.67 μM). These results illustrate that the hybridization of coumarin with various substituted hydrazides with azomethine linkage could afford potential α-glucosidase inhibitors.

The structure–activity relationship (SAR) was also analyzed based on the activity data. Since the Ar-groups attached directly to the acyl part of hydrazones is the sole variation in the structures of synthesized conjugates, the effect of these groups could be the only reason for the range of values of minimum inhibitory concentration.

**Fig. 2:**
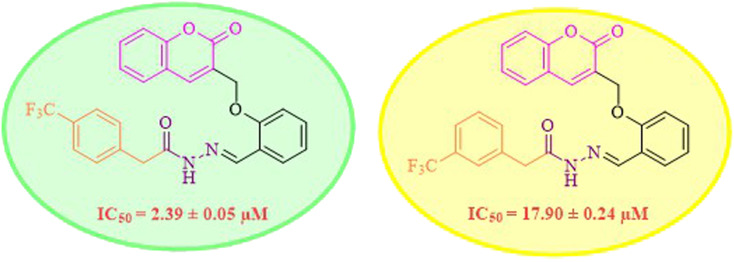
Structure–activity relationship of compound 7c as compared to compound 7i.

While scanning 9 compounds, 7c was found as the most potent inhibitor (IC_50_ = 2.39 ± 0.05 μM). Its structure includes *p*-substituted benzyl moiety as Ar-group. 7i contains *m*-trifluoromethylbenzyl as Ar-group and showed IC_50_ value of 17.90 ± 0.24 μM. Although the substitution on the benzyl part in both compounds is the same, there is a marked difference between the inhibitory potentials of these two derivatives. The high activity of 7c might be due to the presence of electron withdrawing substitution at *para* position of benzyl group (Fig. 2).

Similarly, compounds 7a and 7d bear *o*- and *m*-chloro substituted phenyl rings as Ar-groups. 7a showed IC_50_ value of 4.95 ± 0.17 μM and 7d had IC_50_ value of 3.76 ± 0.14 μM, affirming the importance of position of substitutional groups. On the other hand, the derivative 7h contains trifluoromethyl substitutions at *meta* position of phenyl ring. It showed noteworthy activity with IC_50_ value of 6.14 ± 0.13 μM but this value demonstrates it to be a bit less potent inhibitor as compared to 7a and 7d. Moreover, compound 7g with a *p*-substituted methoxy group was also found to be as highly active as compared to acarbose. It seemed like not only the position but also the nature of substituent is important.

The analog 7f has biphenyl ring and showed good biological activity. Similarly, the derivative 7e with 3-pyridyl and *p*-methoxyphenyl as Ar-group is also highly active. The inactivity of 7b might be due to the non-interaction of its Ar-group substituent with the active site residue of enzyme. These results indicates that the nature as well as position of substituents affects the inhibitory potential of these derivatives. Moreover, molecular docking was performed to understand the binding interactions of these conjugates.

### 2.3. Docking analysis

All the compounds (except 7b) showed excellent inhibitory potential against the α-glucosidase enzyme; therefore, the active hits were docked into the binding cavity of α-glucosidase. The binding mode analysis reflects that the acyl-hydrazone moiety of these compounds is essential for binding with the active site residues including Glu277, Asp352, Asn350, Gln353, Arg213, Tyr347, Thr306, and His351. All the compounds adapt to similar positions in the active site with little conformational differences. The Ar-group of these compounds are resided towards Trp58, Phe301, Tyr347, Glu349, Asn350, while the coumarin ring is lied towards Tyr158, Phe159, Phe178, Val216, and Arg442. Interestingly, the acyl-hydrazone moiety of these molecules is merged in between Arg213, His351, Asp352, and Gln353, which are core residues of α-glucosidase enzyme.

**Fig. 3:**
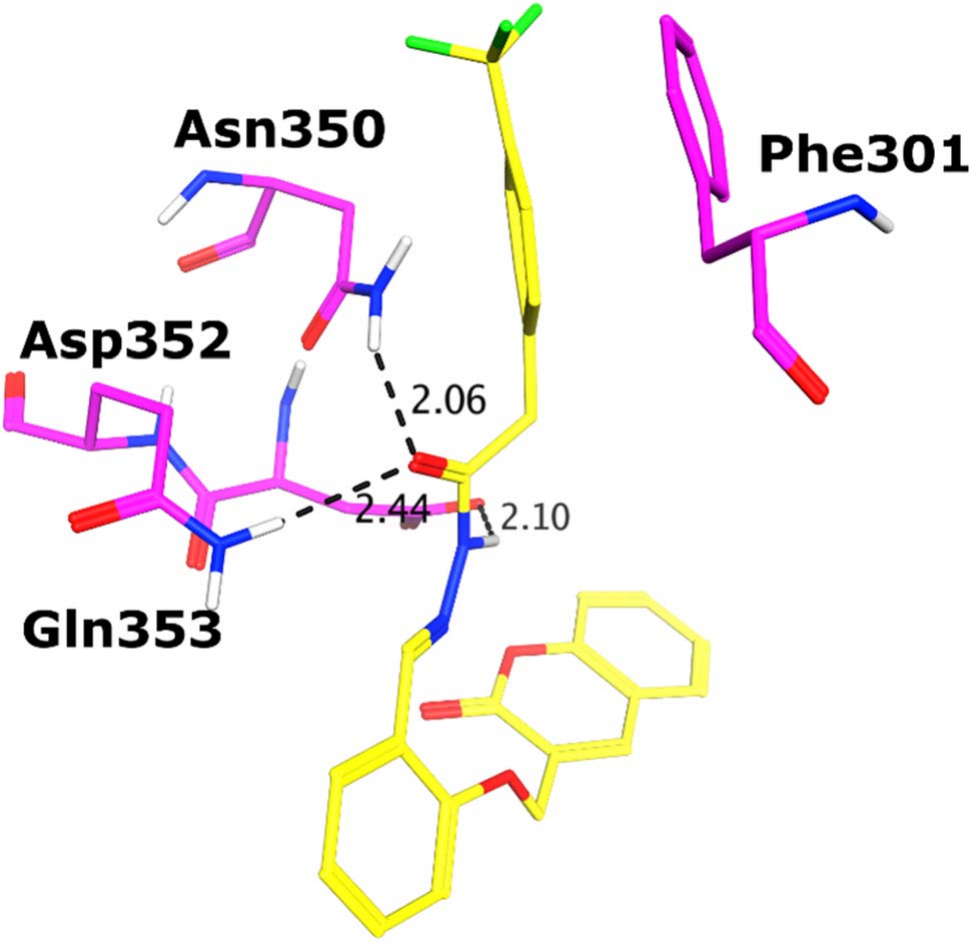
The docked conformation of compound 7c (presented in yellow sticks) is shown in the active site of α-glucosidase. The binding residues in the active site are presented in magenta sticks, H-bonds are depicted in black dotted lines.

The binding poses of the most active compound 7c, showed that its acyl hydrazone group binds with the side chains of Asp352, Asn350 and Gln353 through strong hydrogen bonds (H-bond) (Fig. 3). While the second most active hit, *i.e.*, 7f only mediates a H-bond with the side chain of Tyr347. In addition, the phenyl ring of Phe301 provides hydrophobic interaction to the compounds 7c and 7f. We observed that Glu277, Asn350, and Gln353 provides H-bonds to the acyl-hydrazone group of the compound 7a, while compound 7d is stabilized by Thr306 and Tyr347 through H-bonds. Similarly, Glu277 and Gln353 established H-bonds with the acyl-hydrazone group of 7g. Similarly, the acyl-hydrazone of 7h mediates H-bond with Gln353, while the fluorine of 7h also mediates bidentate interaction with the side chain of Arg213. Like compound 7d, the core hydrazone group of 7i also forms H-bond with Tyr347, moreover, 7i also forms H-bonds with Asn350 and Gln353. While Phe301 provides π–π interactions to compound 7h, and 7i. The binding mode of the least active compound (7e) demonstrates that instead of acyl-hydrazone group of this molecule, the polar atoms in the coumarin ring interact with the His351 and Arg213 through H-bonds. The interactions of all the docked compounds with the active site residues of enzyme are shown in Fig. S1 (ESI†). The docking scores of these compounds are in range of −9.91 to −6.58 kcal mol^−1^, which shows good binding potential of these inhibitors with the active site residues of enzyme, and excellent correlation with the *in vitro* results. The protein–ligand interactions and the docking scores of each docked compound are given in Table 2.

**Table 2:** The protein–ligand interactions and the docking scores of compounds^a^

Compounds	Docking score (kcal mol^−1^)	Protein–ligand interactions			
		Ligand atoms	Receptor atoms	Interaction type	Distance (Å)
7c	−9.91	N6	OD2-ASP352	HBD	2.34
		O5	ND2-ASN350	HBA	2.19
		O5	NE2-GLN353	HBA	2.50
		6-Ring	6-Ring-PHE301	π–π	3.03
7f	−8.81	O19	OH-TYR347	HBA	2.47
		6-Ring	6-Ring-PHE301	π–π	3.13
7d	−8.62	N13	OG1-THR306	HBD	2.52
		O12	OH-TYR347	HBA	1.96
7a	−8.58	N13	OE2-GLU277	HBD	2.48
		O12	ND2-ASN350	HBA	2.23
		O12	NE2-GLN353	HBA	2.09
7g	−8.48	N13	OE2 GLU277	HBD	3.16
		O12	NE2 GLN353	HBA	3.01
7h	−8.39	O12	NE2-GLN353	HBA	2.79
		F18	NH2-ARG213	HBA	2.99
		F19	NH2-ARG213	HBA	2.91
		6-Ring	6-Ring-PHE303	π–π	3.62
7i	−7.82	O5	OH-TYR347	HBA	3.04
		O5	ND2-ASN350	HBA	2.86
		N8	NE2-GLN353	HBA	2.94
		6-Ring	6-Ring-PHE301	π–π	3.99
7e	−6.58	O46	NE2-HIS351	HBA	2.83
		O47	NH2-ARG213	HBA	3.09
Standard (acarbose)	−6.19	O13	OD2-ASP352	HBD	2.72
		O17	OD2-ASP69	HBD	2.58
		O22	OD2-ASP69	HBD	2.63
		O59	OD1-ASP307	HBD	3.41
		O69	O-ASP352	HBD	2.87
		O74	OE2-GLU411	HBD	2.75
		O77	OD1-ASP307	HBD	3.05
		O13	NH2-ARG213	HBA	2.98
		O 13	NE2-HIS351	HBA	2.87
		O15	NE2-HIS351	HBA	3.05
		O17	NH1-ARG442	HBA	2.82
		O59	NE2-GLN353	HBA	2.58
		O64	OH-TYR347	HBA	3.40
		O64	ND2-ASN350	HBA	3.14
		O65	NE2-GLN353	HBA	2.47

a HBA = hydrogen bond acceptor, HBD = hydrogen bond donor.

## 3. Experimental section

### 3.1. Material and methods

All the chemicals and solvents were purchased from commercial suppliers Sigma Aldrich (Germany) and Alfa Acer (China). The solvents *i.e.*, methanol, chloroform and ethyl acetate were of laboratory grade, so these were purified by applying standard protocols of distillation and drying. Commercial reagents were used without further purification. Merck silica gel 60F_254_ 0.2 nm pre-coated aluminum plates were used for TLC. Chromatograms were visualized by UV at 254 and 365 nm. SMP3 (digital melting point) apparatus was used to determine the melting point of compounds. ^1^H-NMR and ^13^C-NMR spectra were obtained by using 600 MHz and 150 MHz Bruker NMR spectrophotometer, respectively, using tetramethyl silane as an internal reference. Chemical shift values were given in ppm. Solvent used for NMR spectroscopy was DMSO-*d*_6_.

### 3.2. General procedure for the synthesis of 2-((2-oxo-2*H*-chromen-3-yl)methoxy)benzaldehyde (3)

The mixture of salicylaldehyde (3 mmol) and dry methanol (10 mL) in a round bottom flask was subjected to stirring for 5 minutes. Methyl acrylate (9 mmol) and triethylamine (3 mmol) were added to the reaction flask. The reaction mixture was stirred at STP. After 5–6 hours, white colored precipitates were formed in flask. The reaction mixture was continued to stir for a total of 15–18 hours. Meanwhile, the progress was monitored by TLC. As the reaction completed, reaction mixture was filtered off. The precipitates obtained of product 3 were washed thoroughly with boiling methanol and then dried. Ultimately, white solid was obtained with melting point 175–178 °C in 87% yield.

### 3.3. General procedure for the synthesis of substituted methyl arylates (5a–i)

Substituted aryl carboxylic acid (4a–i) (5 mmol) was added to methanol (15 mL), followed by the addition of few drops of concentrated sulfuric acid. Reaction mixture was refluxed till completion. On completion of reaction, solvent extraction was performed and then the organic layer was further washed with sodium carbonate solution. Sodium sulfate was used to remove the water contents of organic layer. Low-pressure evaporation yielded solid product.

### 3.4. General procedure for the synthesis of acid hydrazides (6a–i)

Substituted methyl arylate (5a–i) (2.5 mmol) and hydrazine hydrate (10 mmol) were dissolved in methanol (5 mL) and refluxed in a flask for 3–4 hours. Upon completion of reaction (monitored by TLC), the precipitates were obtained by filtration and recrystallized using ethanol.

### 3.5. General procedure for the synthesis of coumarin–hydrazone conjugates (7a–i)

Substituted acid hydrazide (1 mmol) (6a–i) and 2-((2-oxo-2*H*-chromen-3-yl)methoxy)benzaldehyde (1 mmol) (3) were added in dry methanol (10 mL) along with *p*-toluenesulfonic acid in catalytic amount. Reaction mixture was refluxed for 5–6 hours. Reaction completion was monitored by TLC. On completion, the precipitates were filtered off, washed with cold methanol, and dried out at room temperature.

#### 3.5.1. 2-Chloro-*N*′-(2-((2-oxo-2*H*-chromen-3-yl)methoxy)benzylidene)benzohydrazide (7a)

Yellow solid; yield: 60%; mp: 272–275 °C; *R*_f_: 0.50 (90% CHCl_3_/MeOH); ^1^H-NMR (600 MHz, DMSO-*d*_6_) *δ* (ppm): 11.87 (s, 1H, NH), 9.02 (s, 1H, N=CH), 8.53–8.14 (m, 1H, Ar-H), 8.02 (d, 1H, *J* = 7.2 Hz, Ar-H), 7.74 (d, 1H, *J* = 6.6 Hz, Ar-H), 7.62–7.25 (m, 9H, Ar-H), 7.07 (t, 1H, *J* = 6.6 Hz, Ar-H), 5.10 (s, 2H, CH_2_–O); ^13^C-NMR (150 MHz, CDCl_3_) *δ* (ppm): 172.8 (NC=O), 160.2 (C=O), 158.1, 157.7, 153.1, 139.2 (C=N), 131.8, 131.7, 128.6, 128.2, 124.8, 124.7, 124.1, 121.8, 118.9, 116.6, 112.8, 112.6, 65.1; IR (KBr, cm^−1^) *ν*: 3235 (C–H, aromatic), 1711 (C=O, ketone), 1647 (C=O, amide), 1600 (C=C), 1487 (C=N, azomethine); Anal. calcd for C_24_H_17_ClN_2_O_4_; C, 66.60; H, 3.96; N, 6.47; found: C, 66.59; H, 3.95; N, 6.45; MS: *m*/*z* = 433.09 (100.0%) [M + 1].

#### 3.5.2. 1-Cyclopropyl-6-fluoro-4-oxo-*N*′-(2-((2-oxo-2*H*-chromen-3-yl)methoxy)benzylidene)-7-(piperazin-1-yl)-1,4-dihydroquinoline-3-carbohydrazide (7b)

Brown solid; yield: 79%; mp: 275–278 °C; *R*_f_: 0.40 (90% CHCl_3_/MeOH); ^1^H-NMR (600 MHz, DMSO-*d*_6_) *δ* (ppm): 13.05 (s, 1H, NH), 8.95 (s, 1H, N=CH), 8.63 (d, 1H, *J* = 25.8 Hz, Ar-H), 8.29 (s, 1H, Ar-H), 7.88 (d, 1H, *J* = 6 Hz, Ar-H), 7.83 (d, 1H, *J* = 12.6 Hz, Ar-H), 7.78 (d, 1H, *J* = 7.2 Hz, Ar-H), 7.59 (t, 1H, *J* = 7.8 Hz, Ar-H), 7.49–7.47 (m, 2H, Ar-H), 7.40 (d, 1H, *J* = 7.8 Hz, Ar-H), 7.34 (t, 1H, *J* = 7.2 Hz, Ar-H), 7.18 (d, 1H, *J* = 7.8 Hz, Ar-H), 7.12 (d, 1H, *J* = 7.8 Hz, Ar-H), 7.04 (t, 1H, *J* = 7.2 Hz, Ar-H), 5.02 (s, 2H, CH_2_–O), 3.76 (s, 1H, cyclopropyl), 3.45 (m, 4H, piperazinyl), 3.34 (m, 4H, piperazinyl), 1.31 (d, 2H, *J* = 6.6 Hz, cyclopropyl), 1.15 (s, 2H, cyclopropyl); ^13^C-NMR (150 MHz, DMSO-*d*_6_) *δ* (ppm): 174.0 (C=O), 160.8 (NC=O), 159.5 (C=O), 156.4, 153.4, 152.9 (C=N), 146.4 (d, *J*_CF_ = 270 Hz), 143.6, 143.3 (d, *J*_CF_ = 15 Hz), 140.3, 138.05 (d, *J*_CF_ = 75 Hz), 131.7 (d, *J*_CF_ = 30 Hz), 128.8, 128.1, 126.7, 125.5, 124.6, 123.8, 122.7, 121.3 (d, *J*_CF_ = 45 Hz), 118.9, 116.1, 113.2, 111.6, 109.4, 106.6, 65.3, 46.5, 42.8, 40.1, 35.4, 20.8, 7.6; IR (KBr, cm^−1^) *ν*: 3435 (C–H, aromatic), 1721 (C=O, ketone), 1647 (C=O, amide), 1603 (C=C), 1530 (C=N, azomethine); Anal. calcd for C_34_H_30_FN_5_O_5_; C, 67.21; H, 4.98; N, 11.53; found: C, 67.10; H, 4.95; N, 11.45; MS: *m*/*z* = 608.22 (100.0%) [M + 1].

#### 3.5.3. *N*′-(2-((2-oxo-2*H*-Chromen-3-yl)methoxy)benzylidene)-2-(4-(trifluoromethyl) phenyl)acetohydrazide (7c)

White solid; yield: 80%; mp: 262–264 °C; *R*_f_: 0.57 (90% CHCl_3_/MeOH); ^1^H-NMR (600 MHz, DMSO-*d*_6_) *δ* (ppm): 12.02 (s, 1H, NH), 8.83 (s, 1H, N=CH), 8.28 (s, 1H, Ar-H), 8.11 (d, 2H, *J* = 7.8 Hz, Ar-H), 7.90 (d, 3H, *J* = 7.8 Hz, Ar-H), 7.83 (d, 1H, *J* = 7.8 Hz, Ar-H), 7.64 (t, 1H, *J* = 7.2 Hz, Ar-H), 7.46–7.44 (m, 2H, Ar-H), 7.39 (t, 1H, *J* = 7.2 Hz, Ar-H) 7.25 (d, 1H, *J* = 8.4 Hz, Ar-H), 7.09 (t, 1H, *J* = 7.2 Hz, Ar-H), 5.07 (s, 2H, CH_2_–O), 3.32 (s, 2H, COCH_2_); ^13^C-NMR (150 MHz, DMSO-*d*_6_) *δ* (ppm): 161.9 (NC=O), 159.6 (C=O), 156.7, 153.0, 144.3 (C=N), 140.9, 137.3, 132.1, 131.9, 128.7 (d, *J*_CF_ = 15 Hz), 126.4, 125.5, 125.5, 124.8, 124.7 (d, *J*_CF_ = 195 Hz), 121.4, 118.8, 116.2, 113.2, 65.5; IR (KBr, cm^−1^) *ν*: 3230 (C–H, aromatic), 1723 (C=O, ketone), 1649 (C=O, amide), 1601 (C=C), 1543 (C=N, azomethine); Anal. calcd for C_26_H_19_F_3_N_2_O_4_; C, 65.00; H, 3.99; F, 11.86; N, 5.83; found: C, 64.50; H, 3.95; N, 5.79; MS: *m*/*z* = 480.13 (100.0%) [M + 1].

#### 3.5.4. 3-Chloro-*N*′-(2-((2-oxo-2*H*-chromen-3-yl)methoxy)benzylidene)benzohydrazide (7d)

White solid; yield: 90%; mp: 245–248 °C; *R*_f_: 0.53 (90% CHCl_3_/MeOH); ^1^H-NMR (600 MHz, DMSO-*d*_6_) *δ* (ppm): 11.91 (s, NH, 1H), 8.81 (s, N=CH, 1H), 8.28 (s, 1H, Ar-H), 7.96 (s, 1H, Ar-H), 7.88 (t, 2H, *J* = 8.4 Hz, Ar-H), 7.83 (d, 1H, *J* = 7.8 Hz, Ar-H), 7.64 (t, 2H, *J* = 9 Hz, Ar-H), 7.55 (t, 1H, *J* = 7.8 Hz, Ar-H), 7.45 (q, 2H, *J* = 7.8 Hz, Ar-H), 7.39 (t, 1H, *J* = 7.2 Hz, Ar-H), 7.24 (d, 1H, *J* = 8.4 Hz, Ar-H), 7.08 (t, 1H, *J* = 7.2 Hz, Ar-H), 5.07 (s, 2H, CH_2_–O); ^13^C-NMR (150 MHz, DMSO-*d*_6_) *δ* (ppm): 161.6 (NC=O), 159.6 (C=O), 156.7, 153.0, 144.0 (C=N), 140.9, 135.5, 133.3, 132.1, 131.8, 131.6, 130.5, 128.7, 127.4, 126.5, 123.3, 124.8, 123.8, 122.6, 121.4, 118.8, 116.2, 113.2, 65.5; IR (KBr, cm^−1^) *ν*: 3215 (C–H, aromatic), 1719 (C=O, ketone), 1647 (C=O, amide), 1603 (C=C), 1543 (C=N, azomethine); Anal. calcd for C_24_H_17_ClN_2_O_4_; C, 66.60; H, 3.96; Cl, 8.19; N, 6.47; Found: C, 66.58; H, 3.95; N, 6.45; MS: *m*/*z* = 433.09 (100.0%) [M + 1].

#### 3.5.5. *N*′-(2-((2-oxo-2*H*-Chromen-3-yl)methoxy)benzylidene)nicotinohydrazide (7e)

Off-white solid; yield: 83%; mp: 235–238 °C; *R*_f_: 0.50 (90% CHCl_3_/MeOH); ^1^H-NMR (600 MHz, DMSO-*d*_6_) *δ* (ppm): 12.03 (s, NH, 1H), 9.10 (s, N=CH, 1H), 8.80 (t, 2H, *J* = 3.6 Hz, Ar-H), 8.36 (d, 1H, *J* = 7.8 Hz, Ar-H), 8.28 (s, 1H, Ar-H), 7.90 (d, 1H, *J* = 7.8 Hz, Ar-H), 7.83 (d, 1H, *J* = 7.2 Hz, Ar-H), 7.64 (t, 2H, *J* = 7.8 Hz, Ar-H), 7.46 (d, 2H, *J* = 7.2 Hz, Ar-H), 7.41 (t, 1H, *J* = 7.2 Hz, Ar-H), 7.25 (d, 1H, *J* = 8.4 Hz, Ar-H), 7.09 (t, 1H, *J* = 7.8 Hz, Ar-H), 5.07 (t, 2H, *J* = 16.2 Hz, CH_2_–O); ^13^C-NMR (150 MHz, DMSO-*d*_6_) *δ* (ppm): 161.2 (NC=O), 159.6 (C=O), 156.7, 151.1, 147.7, 144.3 (C=N), 140.9, 132.1, 131.9, 129.7, 128.7, 128.1, 126.4, 125.5, 124.8, 124.1, 123.8, 122.4, 121.4, 118.8, 116.2, 113.2, 65.5; IR (KBr, cm^−1^) *ν*: 3237 (C–H, aromatic), 1714 (C=O, ketone), 1647 (C=O, amide), 1602 (C=C), 1544 (C=N, azomethine); Anal. calcd for C_23_H_17_N_3_O_4_; C, 69.17; H, 4.29; N, 10.52; Found: C, 69.15; H, 4.28; N, 10.45; MS: *m*/*z* = 400.12 (100.0%) [M + 1].

#### 3.5.6. *N*′-(2-((2-oxo-2*H*-Chromen-3-yl)methoxy)benzylidene)-1-naphthohydrazide (7f)

White solid; yield: 68%; mp: 270–272 °C; *R*_f_: 0.34 (90% CHCl_3_/MeOH); ^1^H-NMR (600 MHz, DMSO-*d*_6_) *δ* (ppm): 11.97 (s, NH, 1H), 8.74 (s, N=CH, 1H), 8.26–8.22 (m, 2H, Ar-H), 8.07 (d, 1H, *J* = 7.8 Hz, Ar-H), 8.01 (d, 1H, *J* = 7.2 Hz, Ar-H), 7.94 (d, 1H, *J* = 7.8 Hz, Ar-H), 7.77 (dd, 2H, *J* = 20.4 Hz, *J* = 6.6 Hz, Ar-H), 7.60 (m, 4H, Ar-H), 7.40 (m, 2H, Ar-H), 7.34 (t, 1H, *J* = 7.2 Hz, Ar-H), 7.25 (d, 1H, *J* = 8.4 Hz, Ar-H), 7.12 (t, 1H, *J* = 7.2 Hz, Ar-H), 5.05 (s, 2H, CH_2_–O); ^13^C-NMR (150 MHz, DMSO-*d*_6_) *δ* (ppm): 164.6 (NC=O), 159.6 (C=O), 156.7, 152.9, 143.6 (C=N), 140.6, 133.2, 132.9, 132.0, 131.7, 130.5, 130.0, 128.7, 128.4, 127.1, 126.5, 126.4, 125.9, 125.2, 125.0, 124.8, 123.9, 122.7, 121.5, 118.8, 116.2, 113.2, 65.5; IR (KBr, cm^−1^) *ν*: 3238 (C–H, aromatic), 1710 (C=O, ketone), 1647 (C=O, amide), 1600 (C=C), 1480 (C=N, azomethine); Anal. calcd for C_28_H_20_N_2_O_4_; C, 74.99; H, 4.50; N, 6.25; O, 14.27; found: C, 74.95; H, 4.45; N, 6.28; MS: *m*/*z* = 449.14 (100.0%) [M + 1].

#### 3.5.7. 4-Methoxy-*N*′-(2-((2-oxo-2*H*-Chromen-3-yl)methoxy)benzylidene)benzohydrazide (7g)

White solid; yield: 76%; mp: 256–258 °C; *R*_f_: 0.36 (90% CHCl_3_/MeOH); ^1^H-NMR (600 MHz, DMSO-*d*_6_) *δ* (ppm): 11.72 (s, 1H, NH), 8.82 (s, 1H, N=CH), 8.23 (s, 1H, Ar-H), 7.92–7.88 (m, 3H, Ar-H), 7.84 (d, 1H, *J* = 7.2 Hz, Ar-H), 7.64 (t, 1H, *J* = 7.8 Hz, Ar-H), 7.46–7.38 (m, 3H, Ar-H), 7.24 (d, 1H, *J* = 8.4 Hz, Ar-H), 7.08–7.03 (m, 3H, Ar-H), 5.06 (s, 2H, CH_2_–O), 3.82 (s, 3H, methyl); ^13^C-NMR (150 MHz, DMSO-*d*_6_) *δ* (ppm): 162.5 (NC=O), 162.0, 159.6 (C=O), 156.5, 153.0, 142.8 (C=N), 140.7, 132.0, 131.4, 129.6, 128.7, 126.2, 125.5, 124.8, 123.9, 122.9, 121.4, 118.8, 116.2, 113.7, 113.1, 65.4, 55.5; IR (KBr, cm^−1^) *ν*: 3230 (C–H, aromatic), 1712 (C=O, ketone), 1645 (C=O, amide), 1602 (C=C), 1477 (C=N, azomethine); Anal. calcd for; C, 70.09; H, 4.71; N, 6.54; O, 18.67; found: C, 70.08; H, 4.70; N, 6.48; MS: *m*/*z* = 429.14 (100.0%) [M + 1].

#### 3.5.8. *N*′-(2-((2-oxo-2*H*-Chromen-3-yl)methoxy)benzylidene)-3-(trifluoromethyl)benzohydrazide (7h)

White solid; yield: 65%; mp: 235–237 °C; *R*_f_: 0.44 (90% CHCl_3_/MeOH); ^1^H-NMR (600 MHz, DMSO-*d*_6_) *δ* (ppm): 12.03 (s, 1H, NH), 8.83 (s, 1H, N=CH), 8.28–8.23 (m, 3H, Ar-H), 7.93 (d, 2H, *J* = 27.6 Hz, Ar-H), 7.80 (d, 2H, *J* = 34.2 Hz, Ar-H), 7.64 (s, 1H, Ar-H), 7.45–7.39 (m, 3H, Ar-), 7.25 (s, 1H, Ar-H), 7.09 (s, 1H Ar-H) 5.08 (s, 2H, CH_2_–O); ^13^C-NMR (150 MHz, DMSO-*d*_6_) *δ* (ppm): 161.6 (NC=O), 159.6, (C=O), 156.7, 153.0, 144.2 (C=N), 140.9, 134.4, 132.1, 131.9, 129.3 (d, *J*_CF_ = 165 Hz), 128.3, 126.3, 124.8, 124.2, 123.2 (d, *J*_CF_ = 195 Hz), 121.4, 118.8, 116.2, 113.2, 65.5; IR (KBr, cm^−1^) *ν*: 3210 (C–H, aromatic), 1716 (C=O, ketone), 1648 (C=O, amide), 1600 (C=C), 1548 (C=N, azomethine); Anal. calcd for C_25_H_17_F_3_N_2_O_4_; C, 64.38; H, 3.67; N, 6.01; found: C, 64.38; H, 3.65; N, 6.00; MS: *m*/*z* = 467.11 (100.0%) [M + 1].

#### 3.5.9. *N*′-(2-((2-oxo-2*H*-Chromen-3-yl)methoxy)benzylidene)-2-(3-(trifluoromethyl)phenyl)acetohydrazide (7i)

Yellow solid; yield: 72%; mp: 263–265 °C; *R*_f_: 0.33 (90% CHCl_3_/MeOH); ^1^H-NMR (600 MHz, DMSO-*d*_6_) *δ* (ppm): 11.53 (d, ^4^*J* = 125 Hz, 1H, NH), 8.58 (s, 1H, N=CH), 8.47–7.05 (m, 13H, aromatic), 5.07 (m, 2H, CH_2_–O), 4.10 (s, 1H, diastereotopic), 3.66 (s, 1H, diastereotopic); ^13^C-NMR (150 MHz, CDCl_3_) *δ* (ppm): 160.2 (NC=O), 158.1, 156.8 (C=O), 153.2, 139.3 (C=N), 131.7, 128.2, 124.7, 124.0, 121.9, 117.5 (d, *J*_CF_ = 325 Hz), 116.6, 112.8, 112.6, 65.5, 65.1, 65.0, 39.6; IR (KBr, cm^−1^) *ν*: 3210 (C–H, aromatic), 1716 (C=O, ketone), 1648 (C=O, amide), 1600 (C=C), 1548 (C=N, azomethine); IR (KBr, cm^−1^) *ν*: 3210 (C–H, aromatic), 1716 (C=O, ketone), 1648 (C=O, amide), 1600 (C=C), 1548 (C=N, azomethine); Anal. calcd for C_26_H_19_F_3_N_2_O_4_; C, 65.00; H, 3.99; N, 5.83; found: C, 65.00; H, 3.95; N, 5.80; MS: *m*/*z* = 481.13 (100.0%) [M + 1].

### 3.6. α-Glucosidase inhibition assay and statistical analysis

The α-glucosidase inhibition assay was performed by using our previous reported methodology.^47–49^ Experiments were carried out by using phosphate buffer 50 mM, pH. 6.8. Yeast α-glucosidase enzyme (from *Saccharomyces cerevisiae*) was dissolved according to 0.02 U per well, added 20 μL per well and 135 μL per well phosphate buffer as a reaction mixture into 96-well plate. Test samples were dissolved in 7% DMSO and added 20 μL per well, followed by 15 minutes incubation at 37 °C. Substrate *p*-nitrophenyl-α-d-glucopyranoside was added (0.7 mM) 25 μL per well and changes in absorbance were recorded for 30 minutes at 400 nm. Acarbose was used as positive control and DMSO was used as negative control. Excel and the SoftMax Pro package were used as applications to examine the results for biological activity. The following formula was used to determine the % inhibition.1
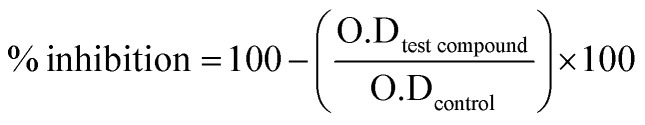


All tested substances' IC_50_ values were calculated using EZ-FIT (Perrella Scientific, Inc., USA). All experiments were carried out in triplicate to reduce the likelihood of mistakes, and differences in the results are reported as Standard Error of Mean Values (SEM).2
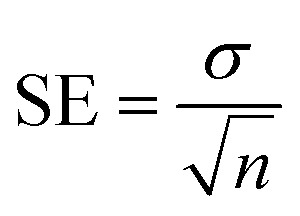


### 3.7. Docking studies

**Fig. 4:**
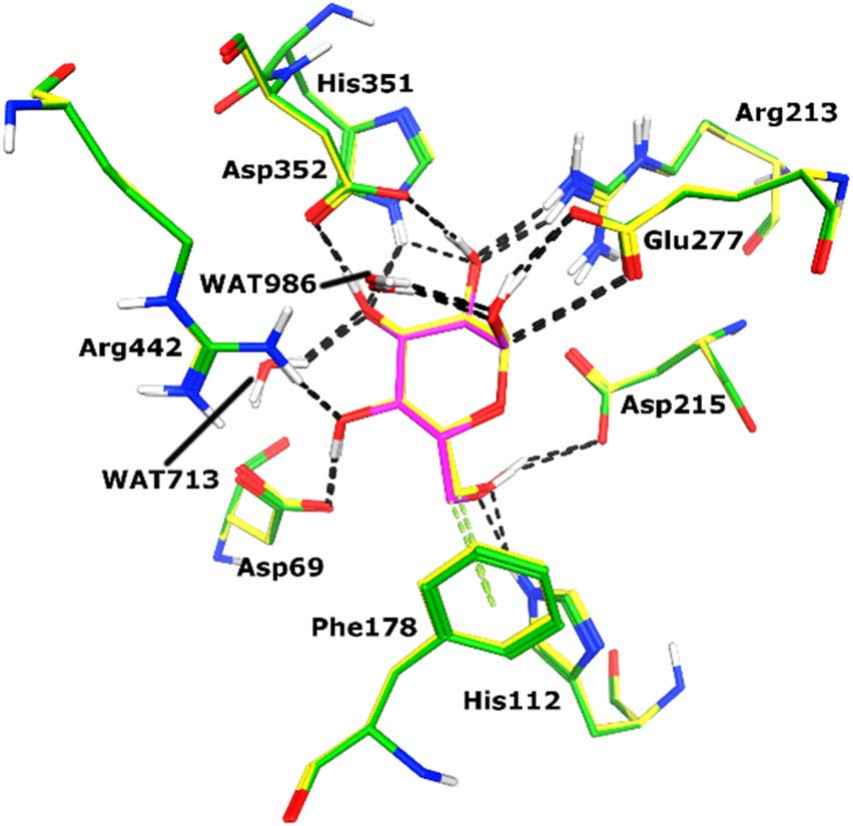
Re-docking results are shown. The docked pose of maltose (shown in magenta sticks) is superimposed on its X-ray conformation (yellow sticks) in the active site of α-glucosidase enzyme with RMSD of 0.56 Å.

Docking of active ligands was carried out on 3A4A, a crystal structure of α-glucosidase bound to a competitive inhibitor called maltose.^50^ For molecular docking, the Molecular Operating Environment (MOE version 2022.02) was used.^51^ Maltose was previously re-docked in the X-ray structure's cognate binding site to test the suitability of the chosen docking procedure. Fig. 4 displays the re-docking data and demonstrates accurate binding of maltose in the α-glucosidase active site with an RMSD of 0.56 Å. Following re-docking analysis, the docking of our ligands was initiated by creating files for the enzyme and ligands. Through the QuickPrep module of MOE, hydrogens were added to 3A4A, and partial charges were applied. The ligands were drawn using MOE, hydrogens, and partial charges were added to the ligands using the MMFF-94x forcefield, and then each structure was minimized using a gradient of 0.1 RMS kcal mol^−1^. After preparing ligands and protein files, docking was conducted by Triangle Matcher docking algorithm and London dG scoring function, and 30 conformations of each ligand were saved.

## 4. Conclusion

A novel series of coumarin–hydrazone conjugates was synthesized, characterized, and evaluated for α-glucosidase inhibitory activity. Except for one molecule, the synthesized derivatives showed remarkable enzyme inhibitory potential which is several folds higher than the standard inhibitor (Acarbose). Among all, the conjugate 7c is the most potent enzyme inhibitor. Different substituted aryl groups attached to the hydrazone side were found to be the main reason for varying inhibitory strength of coumarin–hydrazone conjugates. The molecular docking study affirmed that the synthesized conjugates could be effectively inserted into the active pocket of α-glucosidase. The currently identified biologically active compounds could serve as leads upon further structural optimization in the quest for effective drug candidates for the treatment of diabetes type 2.

## Data availability

Anything related to this article can be obtained from the corresponding authors.

## Conflicts of interest

The authors declare that they have no conflict of interest.

## Supplementary Material

RA-013-D3RA03953F-s001
