# Supplementary material for: Synthesis of novel coumarin–hydrazone hybrids as α-glucosidase inhibitors and their molecular docking studies
Source: RSC Adv. 2023 Sep 4;13(37):26229–38. doi: 10.1039/d3ra03953f (PMC10475976; doi:10.1039/d3ra03953f)
Supplement: RA-013-D3RA03953F-s001 [file RA-013-D3RA03953F-s001.pdf]

Electronic Supplementary Material (ESI) for RSC Advances.
This journal is © The Royal Society of Chemistry 2023

Synthesis of novel coumarin-hydrazone hybrids as α-glucosidase inhibitors and their molecular docking studies

Hafiza Zara Tariq[#a], Aamer Saeed[a*], Saeed Ullah[b,c], Noor Fatima[#a] Sobia Ahsan Halim[b], Ajmal Khan[b], Hesham R El-Seedi [g,h],Muhammad Zaman Ashraf[i], Muhammad Latif[j] , Ahmed Al-Harrasi[b*]

[a] *Department of Chemistry, Quaid-i-Azam University, Islamabad 45320, Pakistan*

[b] *Natural and Medical Sciences Research Center, University of Nizwa, P.O. Box 33, PC 616, Birkat Al Mauz, Nizwa, Sultanate of Oman*

[c] *H.E.J. Research Institute of Chemistry, International Center for Chemical and Biological Sciences, University of Karachi, Karachi-75270, Pakistan*

[g] *School of Food and Biological Engineering, Jiangsu University, Zhenjiang 212013, China -*

[h] *Department of Chemistry, Faculty of Science, Menoufia University, Shebin El-Kom 32512, Egypt*

[i] *Department of Chemistry, Allama Iqbal Open University Islamabad, Pakistan*

[j] *Centre for Genetics and Inherited Diseases (CGID), Taibah University Al–Madinah Al–Munawwarah, Kingdom of Saudi Arabia*

# Both authors contributed equally

*** Address for Correspondence**

Prof. Dr. Aamer Saeed. Email: asaeed@qau.edu.pk, Tel: +92-51-9064-2128

Prof. Dr. Ahmed Al-Harrasi, aharrasi@unizwa.edu.om

^{1}H-NMR and ^{13}C-NMR Spectra of 7a

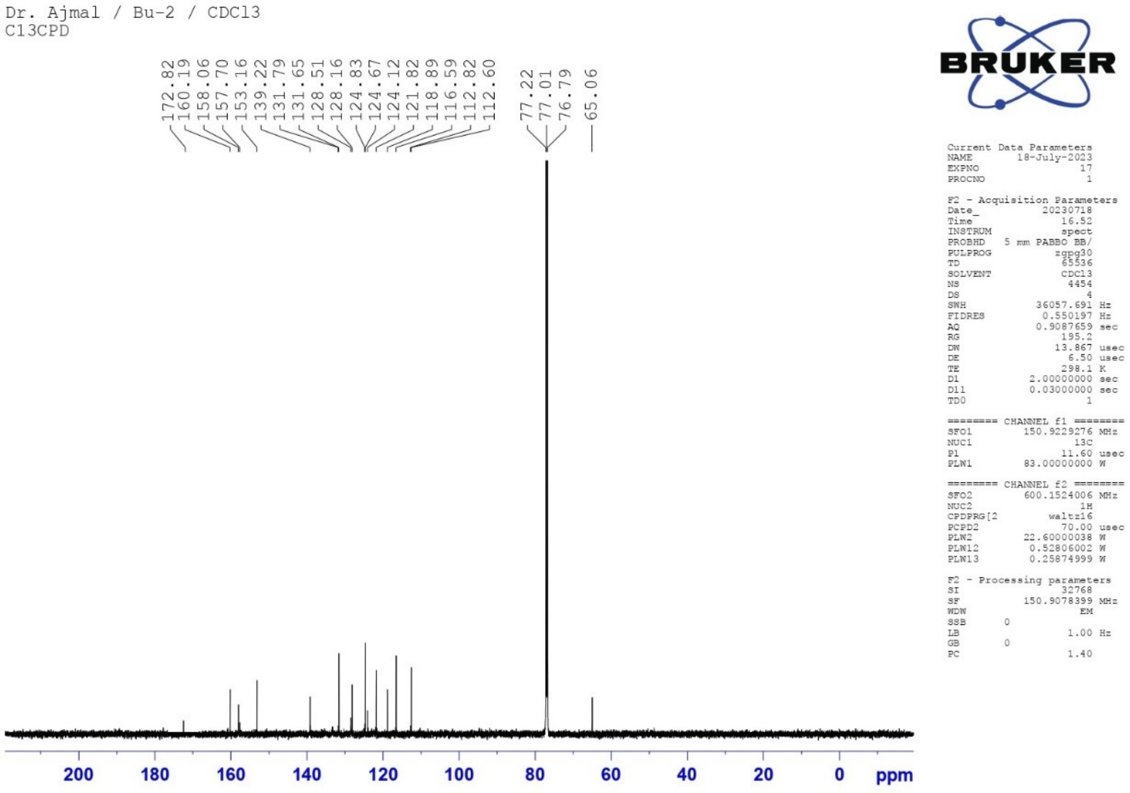

Dr. Ajmal/Bu-2/DMSO
PROTON

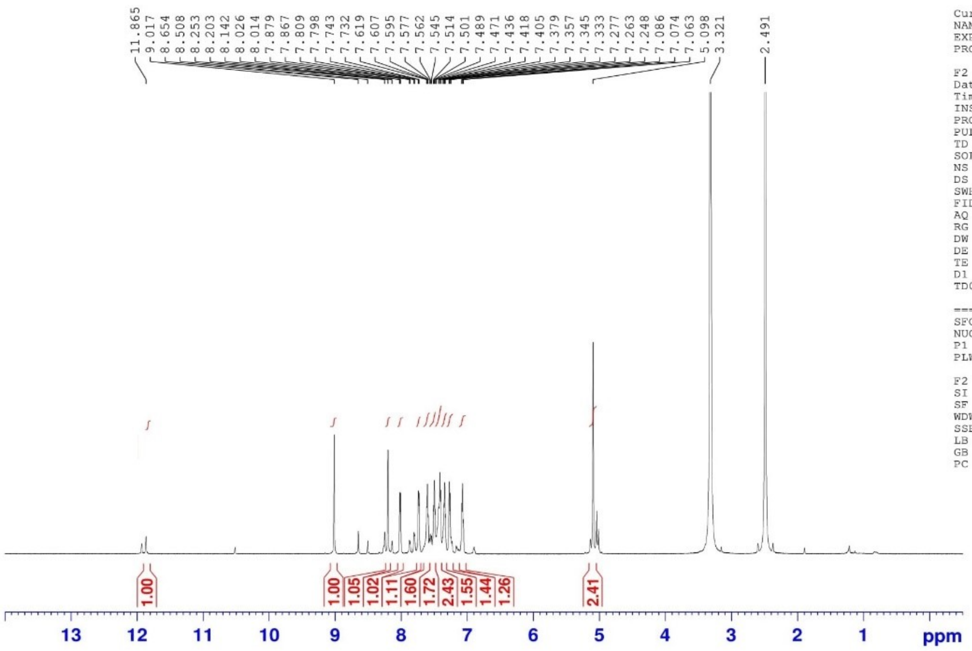


```
Current Data Parameters
NAME      25-July-2021_Dr. Ajmal
EXPNO                     71
PROCNO                     1

F2 - Acquisition Parameters
Date_               20210726
Time                   12.46
INSTRUM                spect
PROBHD   5 mm PABBO BB/
PULPROG                 zg30
TD                     65536
SOLVENT                 DMSO
NS                        16
DS                         2
SWH               12019.230 Hz
FIDRES             0.183399 Hz
AQ                2.7262976 sec
RG                     86.85
DW                    41.600 usec
DE                      6.50 usec
TE                     298.2 K
D1               1.00000000 sec
TD0                        1

======== CHANNEL f1 ========
SFO1            600.1537062 MHz
NUC1                      1H
P1                     10.90 usec
PLW1             22.60000038 W

F2 - Processing parameters
SI                     65536
SF              600.1500107 MHz
WDW                       EM
SSB      0
LB                      0.30 Hz
GB       0
PC                      1.00
```

^{1}H-NMR and ^{13}C-NMR Spectra of 7b

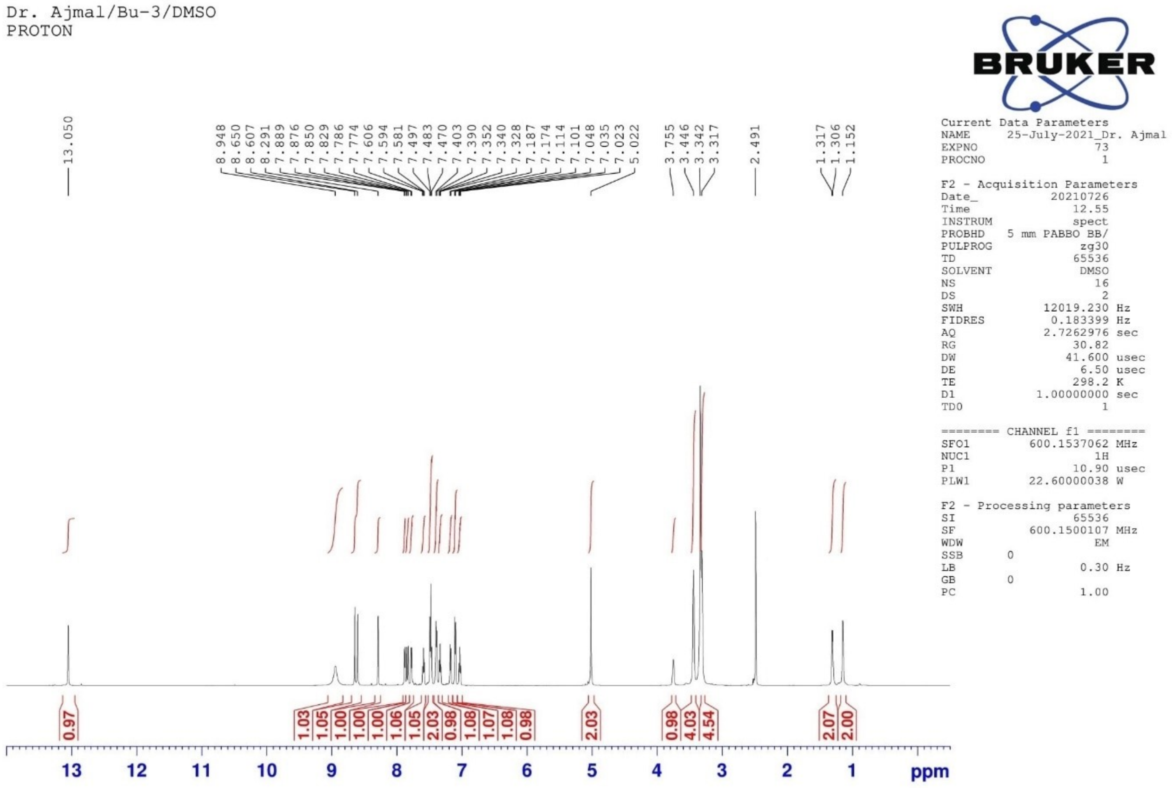

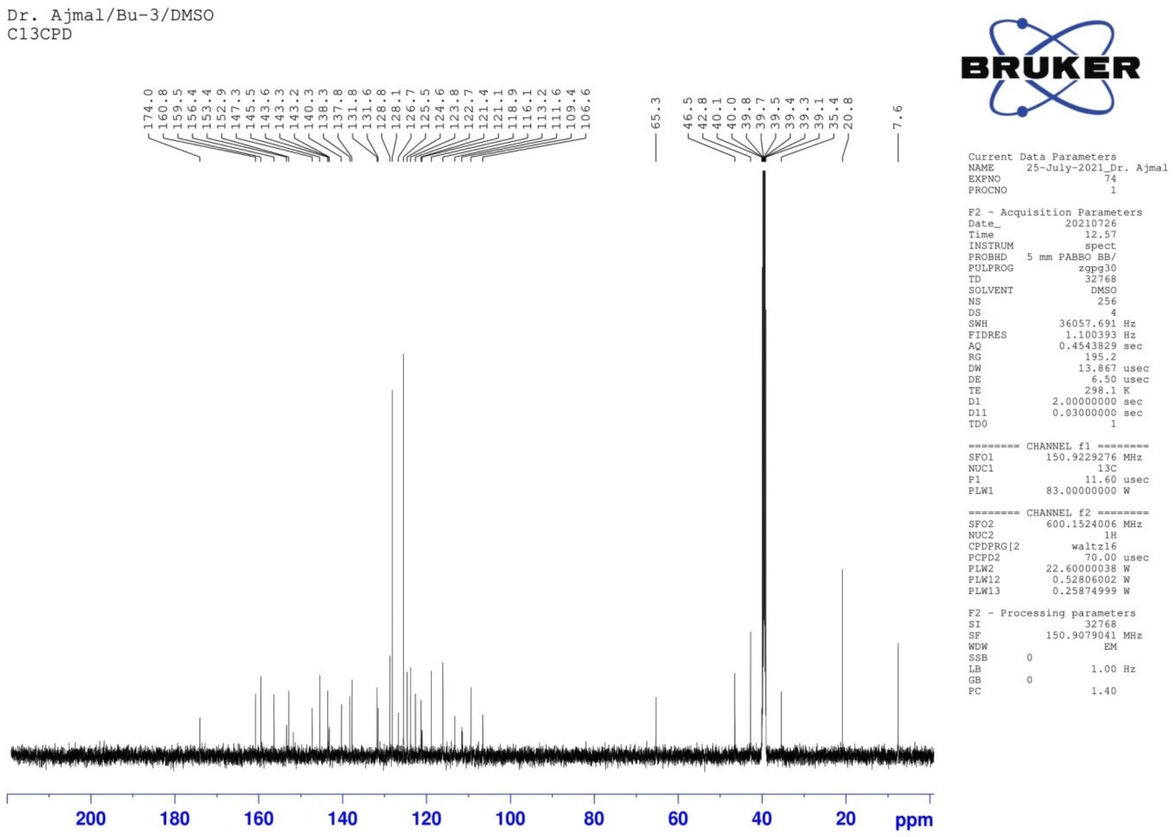

^{1}H-NMR and ^{13}C-NMR Spectra of 7c

Dr. Ajmal/Bu-4/DMSO
PROTON

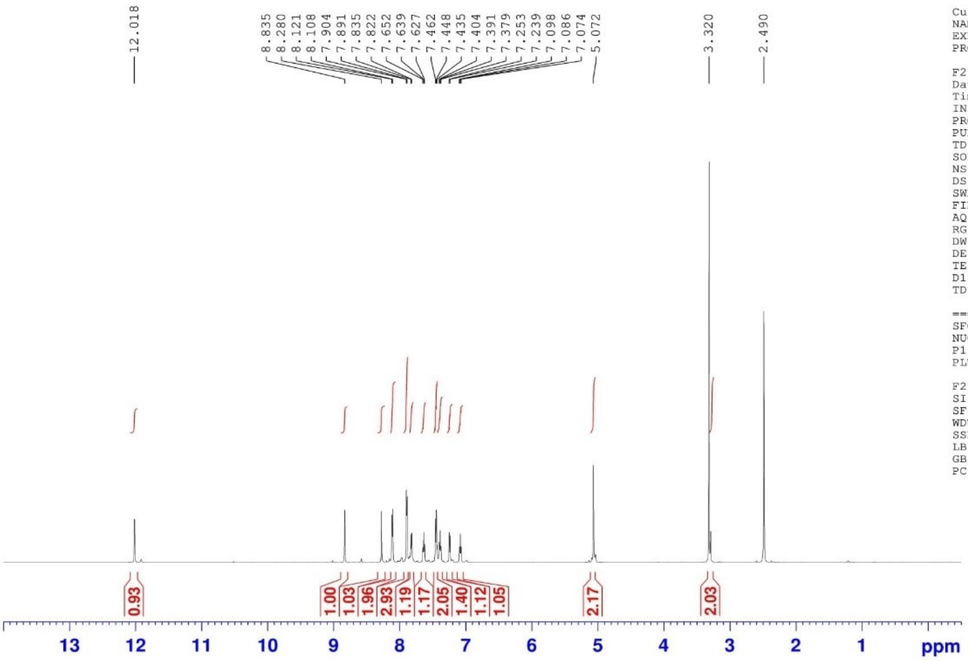

Current Data Parameters
NAME 25-July-2021_Dr. Ajmal
EXPNO 89
PROCNO 1

F2 - Acquisition Parameters
Date_ 20210726
Time 15.35
INSTRUM spect
PROBHD 5 mm PABBO BB/
PULPROG zg30
TD 65536
SOLVENT DMSO
NS 16
DS 2
SWH 12019.230 Hz
FIDRES 0.183399 Hz
AQ 2.7262976 sec
RG 86.85
DW 41.600 usec
DE 6.50 usec
TE 298.1 K
D1 1.00000000 sec
TD0 1

======== CHANNEL f1 ========
SFO1 600.1537062 MHz
NUC1 1H
P1 10.90 usec
PLW1 22.60000038 W

F2 - Processing parameters
SI 65536
SF 600.1500107 MHz
WDW EM
SSB 0
LB 0.30 Hz
GB 0
PC 1.00

Dr. Ajmal/Bu-4/DMSO
C13CPD

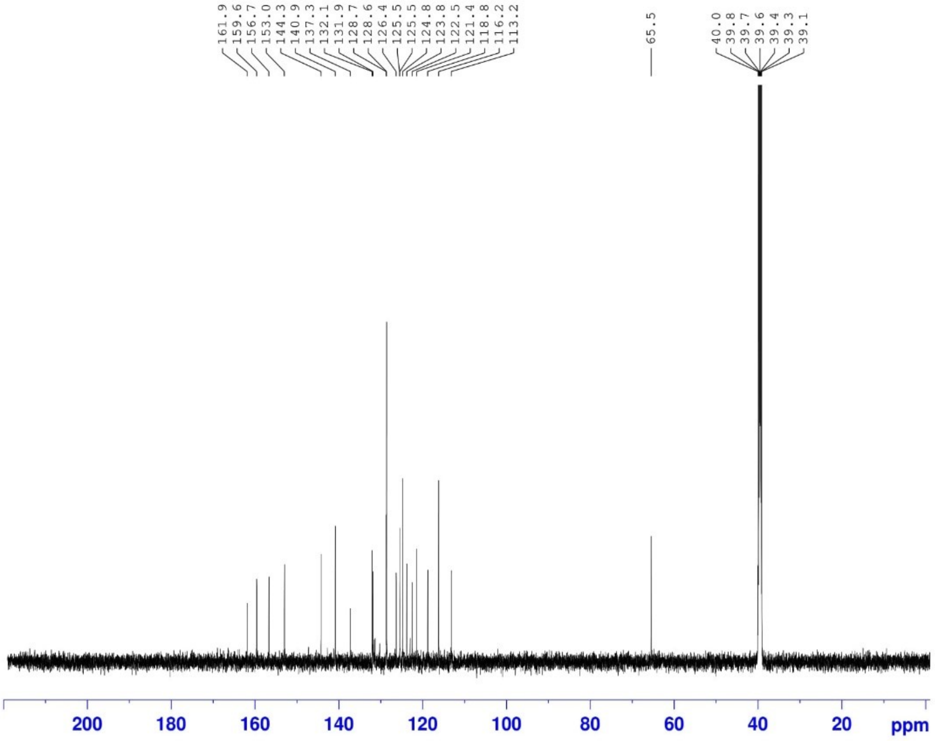

Current Data Parameters

NAME	25-July-2021_Dr. Ajmal	
EXPNO	90	
PROCNO	1	

F2 - Acquisition Parameters

Date_	20210726	
Time	16.18	
INSTRUM	spect	
PROBHD	5 mm PABBO BB/	
PULPROG	zgpg30	
TD	32768	
SOLVENT	DMSO	
NS	1024	
DS	4	
SWH	36057.691	Hz
FIDRES	1.100393	Hz
AQ	0.4543829	sec
RG	195.2	
DW	13.867	usec
DE	6.50	usec
TE	298.2	K
D1	2.00000000	sec
D11	0.03000000	sec
TD0	1	

======== CHANNEL f1 ========

SFO1	150.9229276	MHz
NUC1	13C	
P1	11.60	usec
PLW1	83.00000000	W

======== CHANNEL f2 ========

SFO2	600.1524006	MHz
NUC2	1H	
CPDPRG[2	waltz16	
PCPD2	70.00	usec
PLW2	22.60000038	W
PLW12	0.52806002	W
PLW13	0.25874999	W

F2 - Processing parameters

SI	32768	
SF	150.9079041	MHz
WDW	EM	
SSB	0	
LB	1.00	Hz
GB	0	
PC	1.40	

^{1}H-NMR and ^{13}C-NMR Spectra of 7d

Dr. Ajmal/Bu-7/DMSO
PROTON

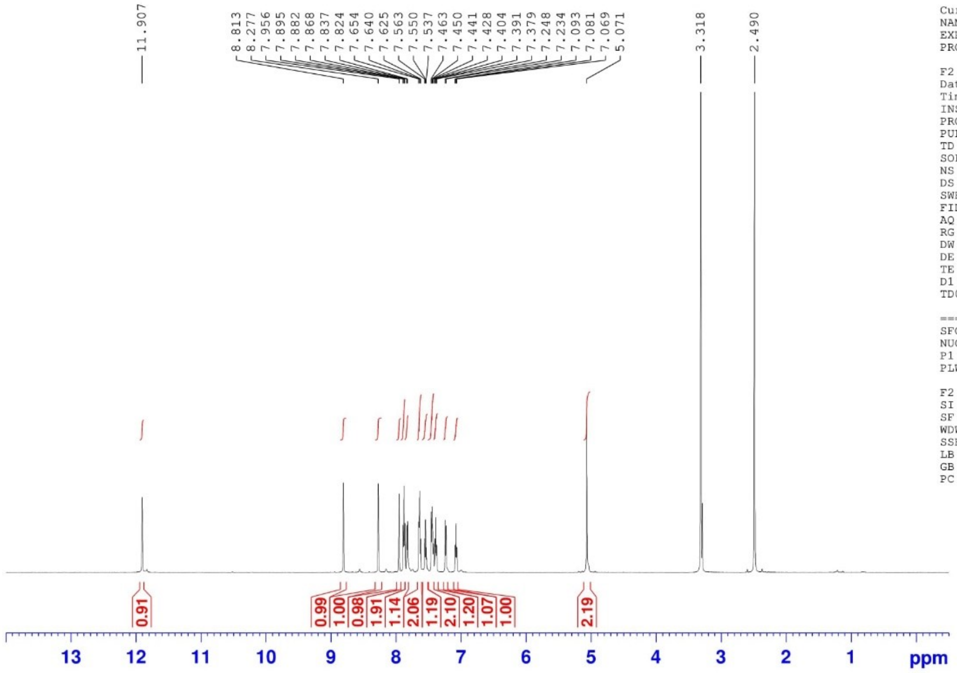


```
Current Data Parameters
NAME      25-July-2021_Dr. Ajmal
EXPNO                     61
PROCNO                     1

F2 - Acquisition Parameters
Date_             20210726
Time                  9.40
INSTRUM              spect
PROBHD   5 mm PABBO BB/
PULPROG               zg30
TD                   65536
SOLVENT               DMSO
NS                      16
DS                       2
SWH              12019.230 Hz
FIDRES            0.183399 Hz
AQ               2.7262976 sec
RG                   86.85
DW                  41.600 usec
DE                    6.50 usec
TE                   298.1 K
D1              1.00000000 sec
TD0                      1

======== CHANNEL f1 ========
SFO1           600.1537062 MHz
NUC1                    1H
P1                   10.90 usec
PLW1           22.60000038 W

F2 - Processing parameters
SI                   65536
SF             600.1500107 MHz
WDW                     EM
SSB      0
LB                    0.30 Hz
GB       0
PC                    1.00
```

Dr. Ajmal/Bu-7/DMSO
C13CPD

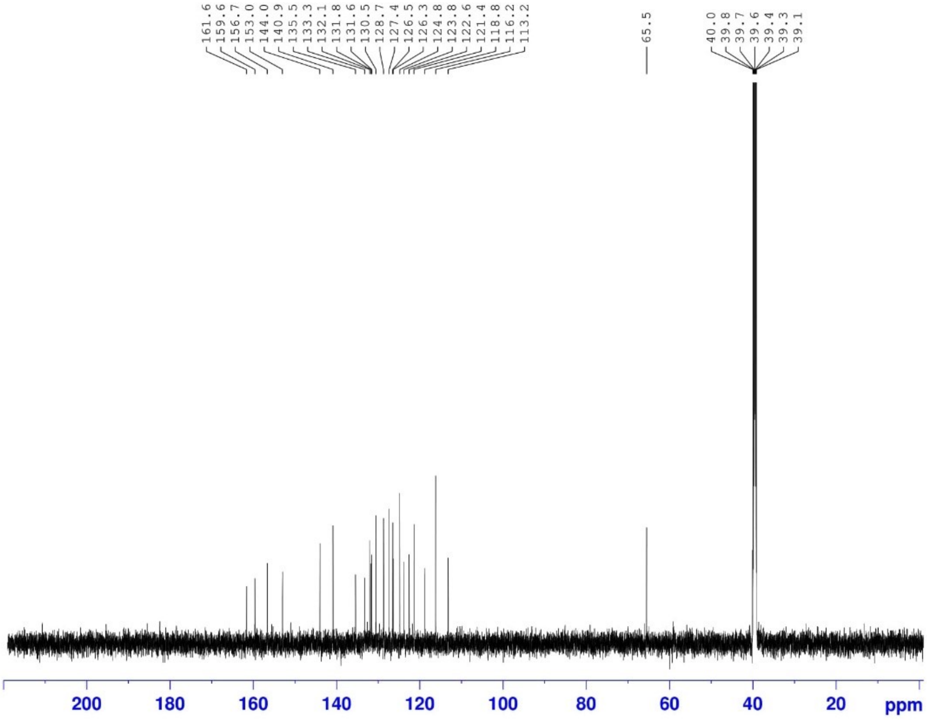

Current Data Parameters
NAME 25-July-2021_Dr. Ajmal
EXPNO 62
PROCNO 1

F2 - Acquisition Parameters
Date_ 20210726
Time 9.44
INSTRUM spect
PROBHD 5 mm PABBO BB/
PULPROG zgpg30
TD 32768
SOLVENT DMSO
NS 512
DS 4
SWH 36057.691 Hz
FIDRES 1.100393 Hz
AQ 0.4543829 sec
RG 195.2
DW 13.867 usec
DE 6.50 usec
TE 298.2 K
D1 2.00000000 sec
D11 0.03000000 sec
TD0 1

======== CHANNEL f1 ========
SFO1 150.9229276 MHz
NUC1 13C
P1 11.60 usec
PLW1 83.00000000 W

======== CHANNEL f2 ========
SFO2 600.1524006 MHz
NUC2 1H
CPDPRG[2 waltz16
PCPD2 70.00 usec
PLW2 22.60000038 W
PLW12 0.52806002 W
PLW13 0.25874999 W

F2 - Processing parameters
SI 32768
SF 150.9079041 MHz
WDW EM
SSB 0
LB 1.00 Hz
GB 0
PC 1.40

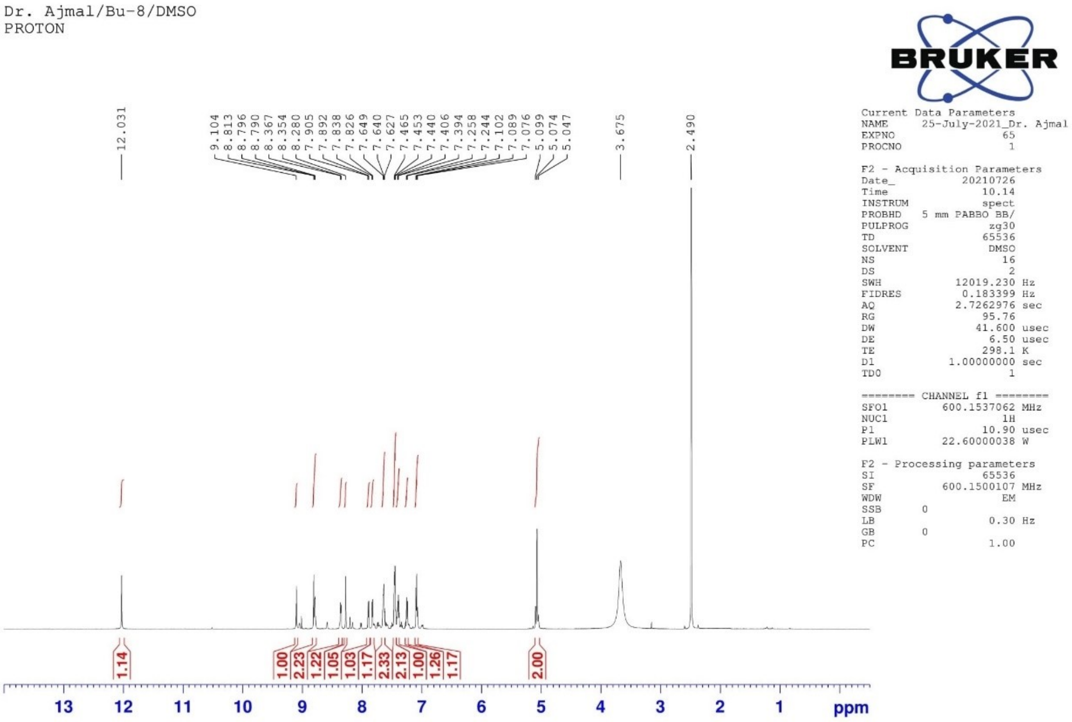

^{1}H-NMR and ^{13}C-NMR Spectra of 7e

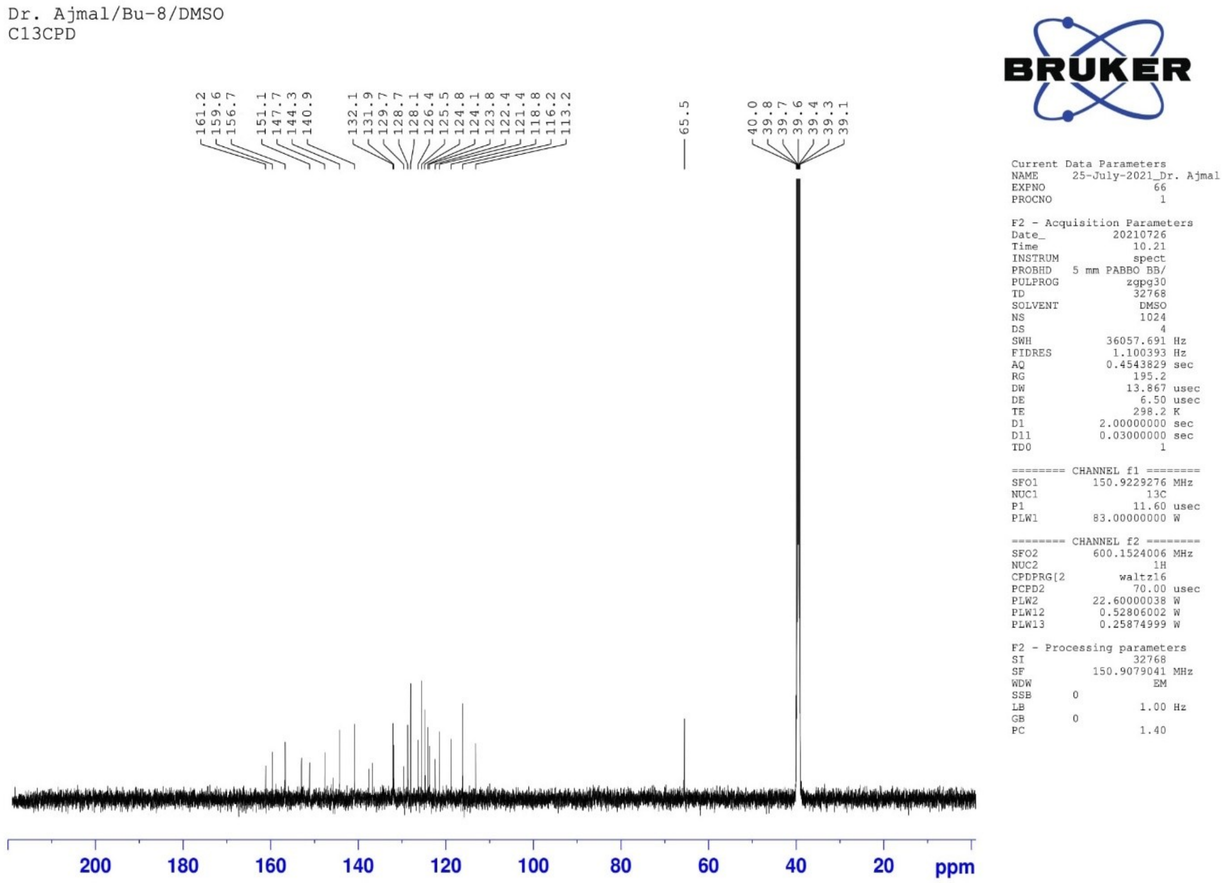

^{1}H-NMR and ^{13}C-NMR Spectra of 7f

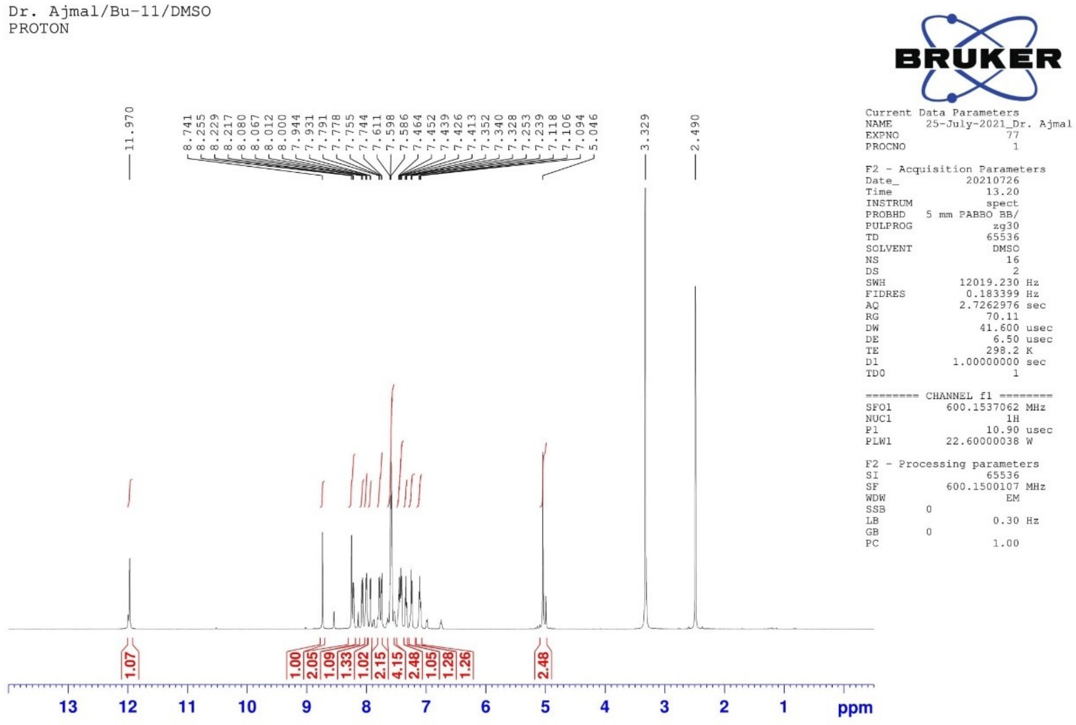

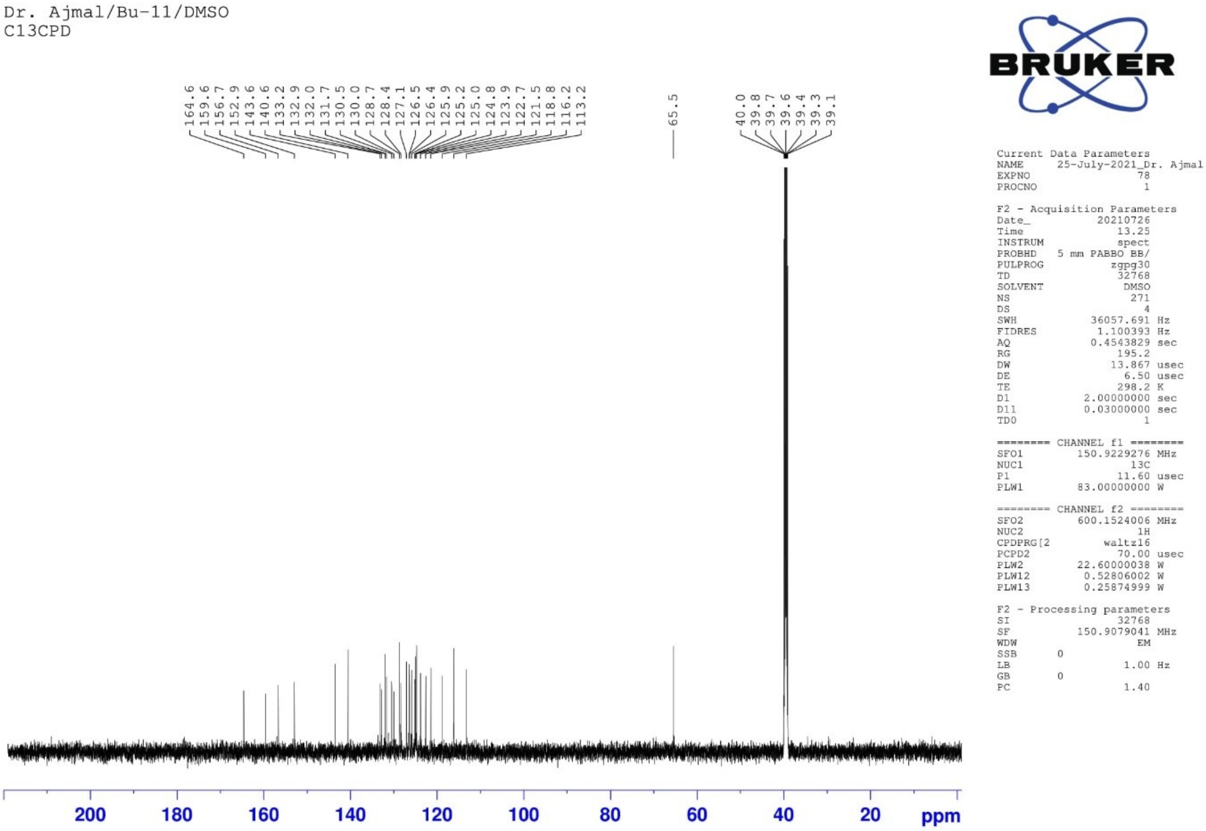

^{1}H-NMR and ^{13}C-NMR Spectra of 7g

Dr. Ajmal/Bu-12/DMSO
PROTON

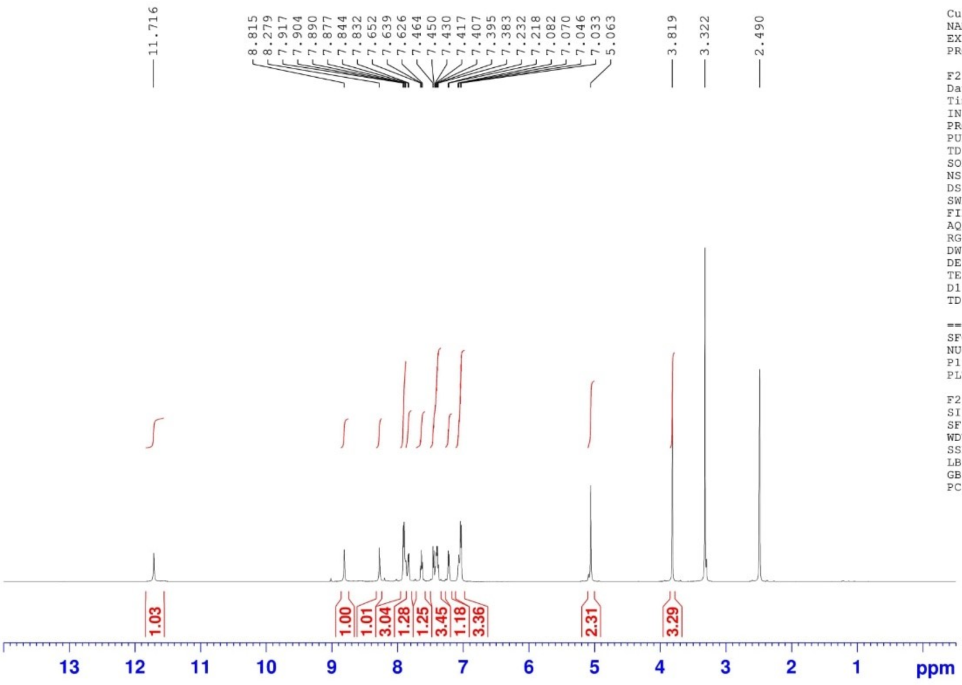


```
Current Data Parameters
NAME      25-July-2021_Dr. Ajmal
EXPNO                 81
PROCNO                 1

F2 - Acquisition Parameters
Date_           20210726
Time               13.42
INSTRUM            spect
PROBHD   5 mm PABBO BB/
PULPROG             zg30
TD                 65536
SOLVENT             DMSO
NS                    16
DS                     2
SWH            12019.230 Hz
FIDRES          0.183399 Hz
AQ             2.7262976 sec
RG                 76.79
DW                41.600 usec
DE                  6.50 usec
TE                 298.2 K
D1            1.00000000 sec
TD0                    1

======== CHANNEL f1 ========
SFO1         600.1537062 MHz
NUC1                  1H
P1                 10.90 usec
PLW1         22.60000038 W

F2 - Processing parameters
SI                 65536
SF           600.1500107 MHz
WDW                   EM
SSB      0
LB                  0.30 Hz
GB       0
PC                  1.00
```

Dr. Ajmal/Bu-12/DMSO
C13CPD

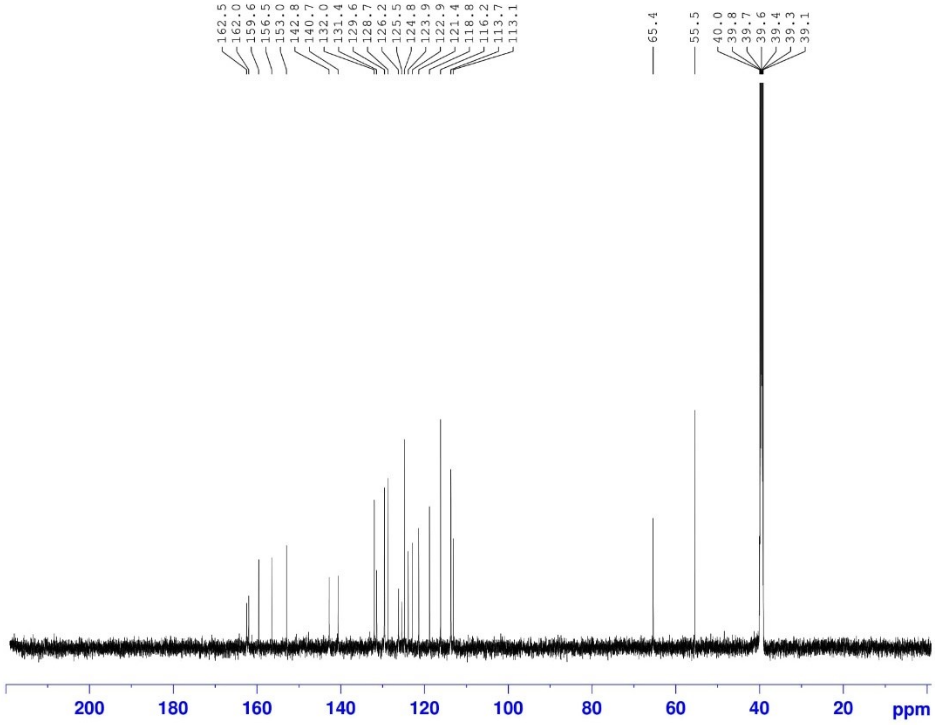


```
Current Data Parameters
NAME     25-July-2021_Dr. Ajmal
EXPNO                    82
PROCNO                    1

F2 - Acquisition Parameters
Date_              20210726
Time                  14.26
INSTRUM               spect
PROBHD   5 mm PABBO BB/
PULPROG             zgpg30
TD                    32768
SOLVENT                DMSO
NS                     1024
DS                        4
SWH              36057.691 Hz
FIDRES            1.100393 Hz
AQ               0.4543829 sec
RG                    195.2
DW                   13.867 usec
DE                     6.50 usec
TE                    298.2 K
D1               2.00000000 sec
D11              0.03000000 sec
TD0                       1

======== CHANNEL f1 ========
SFO1         150.9229276 MHz
NUC1                    13C
P1                    11.60 usec
PLW1          83.00000000 W

======== CHANNEL f2 ========
SFO2         600.1524006 MHz
NUC2                     1H
CPDPRG[2            waltz16
PCPD2                 70.00 usec
PLW2          22.60000038 W
PLW12          0.52806002 W
PLW13          0.25874999 W

F2 - Processing parameters
SI                    32768
SF           150.9079041 MHz
WDW                      EM
SSB      0
LB                     1.00 Hz
GB       0
PC                     1.40
```

^{1}H-NMR and ^{13}C-NMR Spectra of 7h

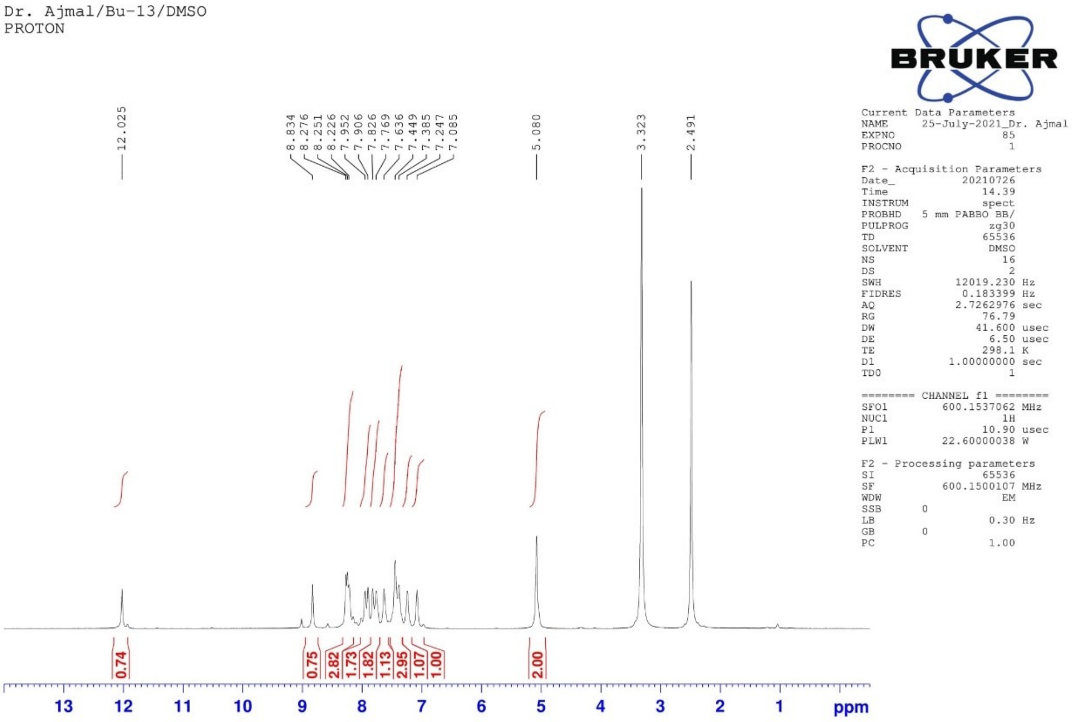

Dr. Ajmal/Bu-13/DMSO
C13CPD

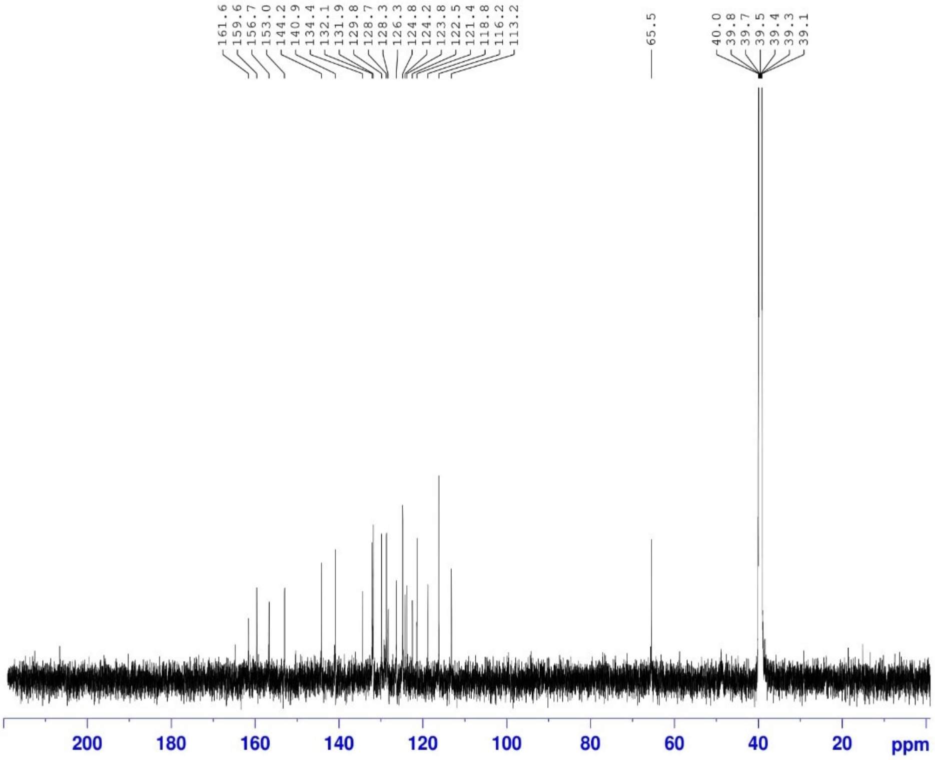


```
Current Data Parameters
NAME     25-July-2021_Dr. Ajmal
EXPNO                 86
PROCNO                 1

F2 - Acquisition Parameters
Date_           20210726
Time               15.22
INSTRUM            spect
PROBHD   5 mm PABBO BB/
PULPROG          zgpg30
TD                 32768
SOLVENT             DMSO
NS                  1024
DS                     4
SWH            36057.691 Hz
FIDRES          1.100393 Hz
AQ             0.4543829 sec
RG                 195.2
DW                13.867 usec
DE                  6.50 usec
TE                 298.1 K
D1            2.00000000 sec
D11           0.03000000 sec
TD0                    1

======== CHANNEL f1 ========
SFO1         150.9229276 MHz
NUC1                 13C
P1                 11.60 usec
PLW1         83.00000000 W

======== CHANNEL f2 ========
SFO2         600.1524006 MHz
NUC2                  1H
CPDPRG[2         waltz16
PCPD2              70.00 usec
PLW2         22.60000038 W
PLW12         0.52806002 W
PLW13         0.25874999 W

F2 - Processing parameters
SI                 32768
SF           150.9079041 MHz
WDW                   EM
SSB      0
LB                  1.00 Hz
GB       0
PC                  1.40
```

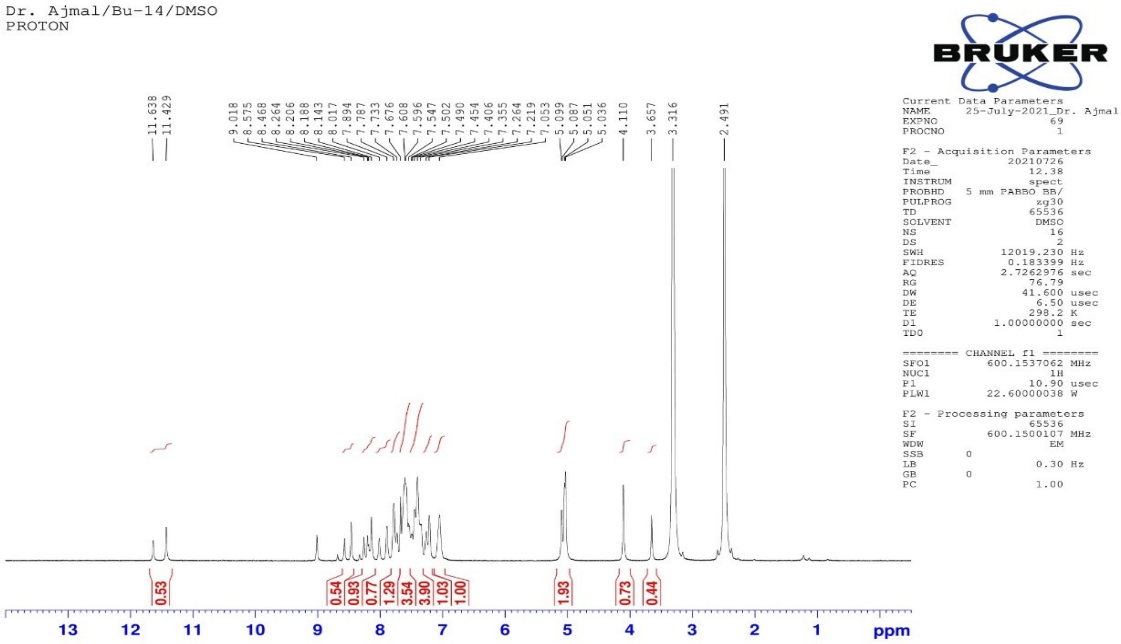

^{1}H-NMR and ^{13}C-NMR Spectra of 7i

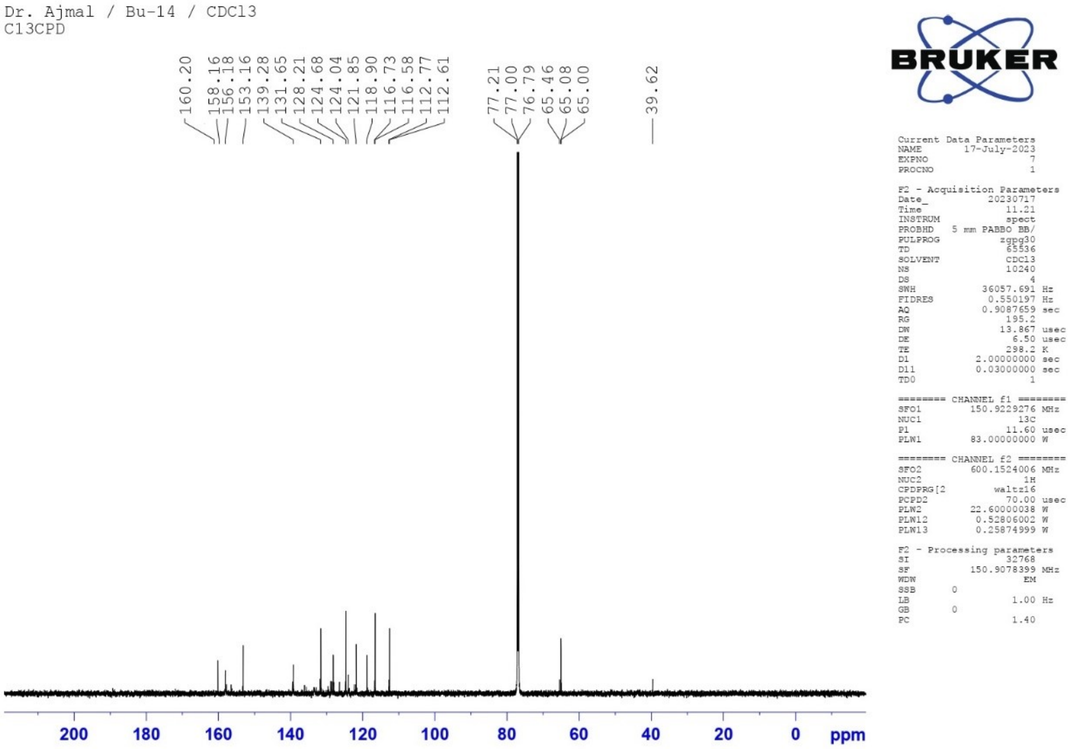

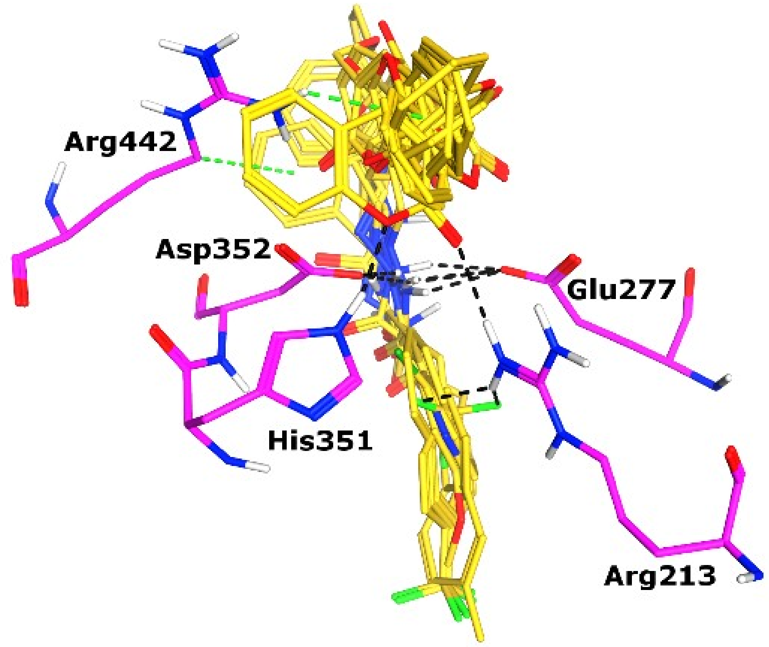

Figure S1. The docked conformations of all the compounds (presented in yellow sticks) are shown in the active site of α-glucosidase. The binding residues in the active site are presented in magenta sticks, H-bonds and hydrophobic bonds are depicted in black and green dotted lines, respectively.
